# Microscopic strain mapping in polymers equipped with non-covalent mechanochromic motifs†

**DOI:** 10.1039/d3mh00650f

**Published:** 2023-06-14

**Authors:** Hanna Traeger, Derek Kiebala, Céline Calvino, Yoshimitsu Sagara, Stephen Schrettl, Christoph Weder, Jess M. Clough

**Affiliations:** a Adolphe Merkle Institute, University of Fribourg Chemin des Verdiers 4 1700 Fribourg Switzerland jessica.clough@unifr.ch; b Cluster of Excellence livMatS, University of Freiburg Georges-Köhler-Allee 105 D-79110 Freiburg Germany; c Department of Materials Science and Engineering, Tokyo Institute of Technology 2-12-1, Ookayama, Meguro-ku Tokyo 152-8552 Japan; d Technical University of Munich, TUM School of Life Sciences Maximus-von-Imhof-Forum 2 85354 Freising Germany

## Abstract

The mechanical failure of polymers remains challenging to understand and predict, as it often involves highly localised phenomena that cannot be probed with bulk characterisation techniques. Here, we present a generalisable protocol based on optical microscopy, tensile testing, and image processing that permits the spatially resolved interrogation of mechanical deformation at the molecular level around defects in mechanophore-containing polymers. The approach can be applied to a broad range of polymeric materials, mechanophores, and deformation scenarios.

## Abstract 2

We present a generalisable methodology based on fluorescent non-covalent mechanophores for producing quantitative maps of local strain around defects within three-dimensional polymeric matrices.

## Graphical abstract

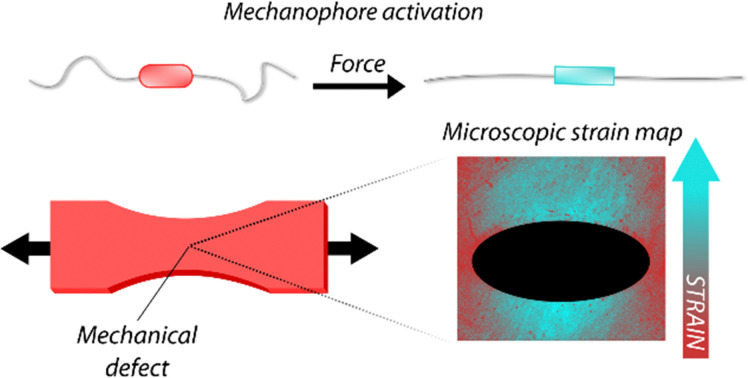

**New concepts**

Mechanochromic probes, which change their absorbance and/or fluorescence in response to mechanical deformation, have attracted considerable interest as tools to monitor mechanical events in polymeric materials, because they enable the molecular-level effects of mechanical force within three-dimensional samples to be monitored *in situ via* a simple spectral or optical readout. However, the exploitation of mechanochromic materials for the quantitative mapping of local strains and stresses has been limited so far. Here, we developed a new methodology that allows fluorescence microscopy images of defect-containing samples to be converted into maps that quantify complex spatial strain distributions. We first demonstrated the method with three different mechanochromic sensing platforms on samples containing macroscopic circular defects, and subsequently validated the approach for more complex strain distributions in a polymer matrix containing inorganic microparticles, highlighting the general applicability of the method. The strain maps reveal surprisingly large deviations in local strain from the externally applied strain in the vicinity of defects, which are known to lead to the permanent damage and catastrophic failure of polymeric materials. With the recent, significant advances in the development of optical probes for mechanical deformation in polymers, we expect that this method will become broadly useful and enhance the quantitative insights that can be gained from mechanochromic sensing systems.

## Introduction

Understanding the effect of defects in polymeric materials under mechanical force is crucial for the prevention of catastrophic materials failure, given that such defects typically generate highly heterogeneous stress and strain distributions that can lead to crack initiation and propagation.^1–4^ Recently, mechanochromic mechanophores have emerged as valuable tools to visualise and investigate these mechanical processes in polymers.^5^ When such motifs are incorporated into a polymeric material and subjected to mechanical force, a weak bond is broken resulting in a change in colour or fluorescence.^1,6–8^ In particular, mechanophores in which the weak bond is covalent in nature have attracted significant research interest as powerful tools for imaging stress and strain distributions in polymeric materials, because they enable the local chemical effects (*i.e.* bond scission) of mechanical force to be monitored *in situ via* a simple spectral or optical readout.^9–19^ Moreover, their response can be spatially resolved, for example, with fluorescence microscopy, to investigate the local mechanical properties of materials, which are inaccessible to bulk characterisation techniques. However, large molecular-level forces, typically in the nanonewton range, are required to overcome the characteristic activation threshold for scission of the covalent bond in covalent mechanophores. Such motifs are therefore less suitable for investigating mechanical processes at low stresses and strains that do not involve significant bond scission,^6^ and ultimately for detecting the onset of damage and crack initiation.^20^ Moreover, covalent mechanophores usually exhibit irreversible mechano-activation, which makes them less useful for repeated strain detection.^21–23^

In addition to mechanophores that operate *via* covalent bond rupture, motifs featuring non-covalent, or supramolecular, dye interactions have been used in recent years to develop new types of mechanochromic materials.^24^ A wide variety of strategies has been explored to render polymeric materials mechanochromic without breaking covalent bonds, for example, the (dis)assembly of excimers^25^ or charge-transfer pairs,^26,27^ displacement of mechanically interlocked dye molecules,^28–31^ or conformational changes in small molecules^32,33^ and macromolecules.^34^ Many of these materials offer the advantage of being able to report on deformation over a much wider deformation range, as the supramolecular interactions in these systems can be broken by smaller molecular-level forces compared to the covalent bond in covalent mechanophores.^24^ However, while significant effort has been devoted to the development of non-covalent mechanophores, they have been little explored for visualising the highly localised phenomena that play a crucial role in the mechanical failure of polymers. In the few examples in which non-covalent mechanophores have been applied to this problem,^32,33,35–38^ the optical or spectral output of the mechanophore was generally not converted to local strain or stress values, so that only relative stress or strain distributions could be detected. In other words, these approaches permitted the identification of areas in which the local stress or strain is relatively greater than in others, but not the determination of absolute values of stress or strain in those areas, which would be valuable for predicting material failure. Furthermore, in some cases, the mechanochromic sensor was coated onto polymeric objects, limiting the insights that could be gained about bulk deformation processes.^37,38^ In other studies, sensor motifs were employed that could not easily be applied to different matrices^39^ or for which the sensing range was intrinsically limited.^4^ In addition to mechanochromic materials, other techniques such as digital image correlation (DIC) and photoelasticity may be used to map local strains quantitatively, but they are generally complex to implement. For example, DIC requires the addition of tracer particles to the matrix whose displacement can be tracked while the sample is mechanically deformed. Moreover, the addition of such particles can alter the mechanical properties of the matrix at the microscopic length scale.^40,41^ Local stresses can also be calculated using birefringence patterns from polarised optical microscopy, though such a calculation is not trivial, particularly in three-dimensional samples.^42–46^

**Fig. 1:**
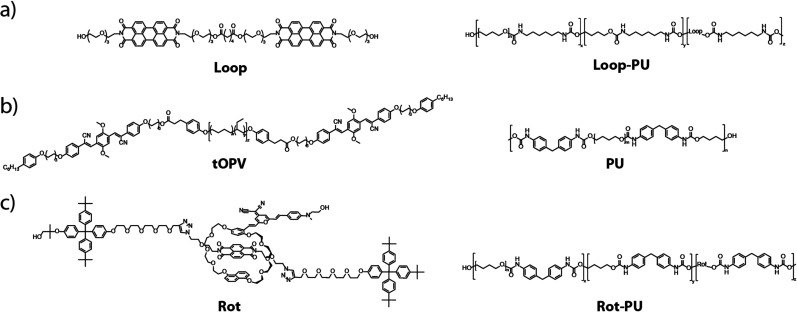
Chemical structures of the three mechanophores investigated in this work, as well as the polymers into which they were incorporated. (a) Loop-forming perylenediimide-based mechanophore (Loop) and the Loop-containing polyurethane (Loop-PU) based on hexamethylene diisocyanate, butane diol (BDO), and poly(tetrahydrofuran) (poly(THF)). (b) Telechelic cyano-OPV-functionalised poly(ethylene-*co*-butylene) (tOPV) and a polyurethane (PU) based on methylene diphenyl diisocyanate (MDI), BDO, and poly(THF). (c) Rotaxane-based mechanophore (Rot) and the Rot-containing polyurethane Rot-PU based on MDI, BDO, and poly(THF).

In this study, we present a simple and generalisable protocol based on optical microscopy, tensile testing, and image processing to generate maps of local strain distributions around defects in mechanochromic polymers. We implement the approach with elastic polyurethanes containing three different supramolecular mechanophores (Fig. 1) previously reported by us,^29,47–50^ which offer sensitive and reversible optical reporting of deformation over a wide strain range. These previous articles all contain emission spectra, colour photos, and videos showing the optical response of the different polymers that we employ here to mechanical deformation. However, the scope of these earlier studies was limited to the characterisation of mechanically induced optical changes at the macroscopic level. Importantly, their mechanochromic responses to defects at the micro- and nanoscopic length scales, where catastrophic fracture initiates, remain unexplored. In the present manuscript, we establish a general imaging protocol that enables the characterisation of complex, spatially varying strain distributions around microscopic defects with mechanochromic materials. To demonstrate the methodology, we introduce circular defects into the mechanochromic polyurethane films and subject them to uniaxial tension, while recording the spectral changes *in situ* with a fluorescence microscope. By calibrating the mechanophore's spectral response against the externally applied strain, we show that it is possible to transform the micrographs into maps of local strain. Furthermore, we showcase the potential of our strain-mapping approach for imaging non-trivial strain distributions in real materials at shorter, microscopic length scales. To this end, we investigated strain distributions in polyurethanes containing inorganic microparticles with confocal microscopy. We show that the creation of microscopic strain maps permits complex deformation patterns to be analysed in these materials. The approach put forward in this work is general and in principle can easily be applied to a broad range of polymeric materials, mechanophores, and mechanical deformation scenarios.

## Results and discussion

We first sought to establish our strain-mapping methodology using the recently reported Loop mechanophore (Fig. 1(a)), which contains two fluorescent perylene diimide (PDI) moieties connected by a short spacer.^47,48^ In the absence of external forces, attractive interactions between the PDIs cause the mechanophore to form a loop and to display predominantly excimer emission. Mechanical deformation applied to the polymer causes the loop structure to unfold and the monomer emission to increase with respect to the excimer emission. Thus, the fluorescence emission colour of the motif changes from orange (excimer) to green (monomer) upon mechanical activation, which can be observed by the unassisted eye under UV light (Fig. S1, ESI†).^47,48^ The mechano-response can also be characterised spectroscopically. In our previous work, the mechano-response at the macroscopic length scale was investigated with conventional fluorescence spectroscopy, in which the signal was acquired from a relatively large area of the sample (spot diameter *ca.* 5 mm).^47,48^ Here, we demonstrate that fluorescence microscopy can be used to quantitatively characterise spatial variations in the optical response at the microscopic length scale. For the purposes of this study, the Loop motif was synthesised and covalently incorporated into a thermoplastic polyurethane elastomer (Loop-PU, Fig. 1(a)) based on hexamethylene diisocyanate (HDI), butane diol (BDO), and poly(tetrahydrofuran) (poly(THF)) at a concentration of 0.075 wt%, as reported before (see ESI† for details and Fig. S2 for TGA characterisation).^48^ Uniform Loop-PU films with a thickness of *ca.* 200 μm were subsequently prepared by compression-moulding.

**Fig. 2:**
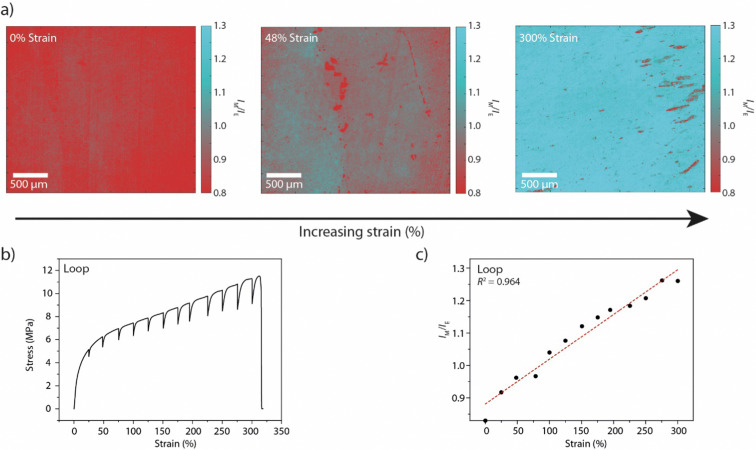
(a) *I*_M_/*I*_E_ maps of films of Loop-PU. The samples are shown in the unstretched state (left, 0% applied strain) and after deformation to 48% (middle) and 300% strain (right). (b) Stress–strain curve of a Loop-PU film. The sample was uniaxially deformed with a strain rate of 50% min^−1^. The elongation was paused at intervals of *ca.* 25% strain for 1–2 minutes to record the fluorescence microscopy images from which the data sets shown in (a), (c), and Fig. S2 (ESI†) were generated. (c) Calibration curve correlating *I*_M_/*I*_E_ to externally applied macroscopic strain. The average values of *I*_M_/*I*_E_ were calculated from the corresponding *I*_M_/*I*_E_ maps of Loop-PU (Fig. 2(a) and Fig. S2, ESI†).

With the Loop-PU films in hand, we monitored optical changes in homogeneous Loop-PU films while subjecting them to different levels of uniaxial strain using a setup comprised of a microtensile tester and a fluorescence microscope (see ESI† for details). We generated maps of the local monomer-to-excimer emission intensity ratio, *I*_M_/*I*_E_, (Fig. 2(a) and Fig. S3, ESI†) from fluorescence micrographs by the following procedure. The films were uniaxially deformed at a strain rate of 50% min^−1^ to a maximum strain of *ca.* 300%. The deformation was paused at strain intervals of 25% for 1–2 minutes in order to acquire fluorescence microscopy images, during which time some stress relaxation occurs (Fig. 2(b)). At each strain step, a pair of fluorescence microscopy images was acquired at a magnification of 5×, using filters that permit the selective transmission of light originating from monomer (*λ*_ex_: 480/40 nm; *λ*_em_: 535/50 nm; numbers indicate the wavelength of peak transmission and the full width at half maximum, respectively) and excimer (*λ*_ex_: 469/35 nm; *λ*_em_: 620/52 nm) species. The images were converted into 1360 × 1024 arrays in which each element represents the fluorescence intensity from a sample area of 2 × 2 μm (see ESI† for details). The division of the corresponding elements of matrix pairs (obtained from image pairs showing monomer and excimer emission at the same strain) afforded matrices whose elements represent the monomer-to-excimer emission intensity ratio, *I*_M_/*I*_E_, as a function of location and for a given externally applied strain. These matrices can then be graphically displayed as maps showing *I*_M_/*I*_E_ as a function of location in the sample (Fig. 2(a) and Fig. S3, ESI†). These maps contain large amounts of quantitative data (one value of *I*_M_/*I*_E_ per pixel), which are displayed as pseudo-colour images for the convenience of visual inspection. We note that it is more straightforward to calculate *I*_M_/*I*_E_ values from intensity values in fluorescence images than from changes in RGB colour values in colour photographs, given that the relationship between RGB values and spectral data is not trivial.^51,52^ An inspection of *I*_M_/*I*_E_ maps acquired at different strains shows that *I*_M_/*I*_E_ increases rather uniformly across the field of view, as a result of strain-induced unfolding of the Loop mechanophores. The maps also reveal small defects, around which the *I*_M_/*I*_E_ value deviates from the mean. A calibration curve was obtained by plotting the average *I*_M_/*I*_E_ values extracted from a series of maps against the externally applied strain (Fig. 2(c)). In this plot, the *I*_M_/*I*_E_ values show a linear dependence on the applied strain. A comparison of this data with the ratio of monomer and excimer emission intensities acquired by conventional fluorescence spectroscopic measurements on macroscopic samples as a function of strain^48^ demonstrates that the microscopically detected *I*_M_/*I*_E_ are directly correlated with macroscopic mechanoresponses established by conventional techniques (Fig. S4, ESI†). For both techniques, the monomer-to-excimer emission intensity ratio is a linear function of the applied strain. The fact that the slopes of the respective functions (0.0013 and 0.0018) are nearly identical is serendipitous, as these functions depend on the specific acquisition parameters. Nevertheless, the fact that *I*_M_/*I*_E_ and strain are linearly correlated in both cases reflects that macroscopic spectroscopic measurements can be substituted by imaging techniques that additionally provide spatial resolution.

**Fig. 3:**
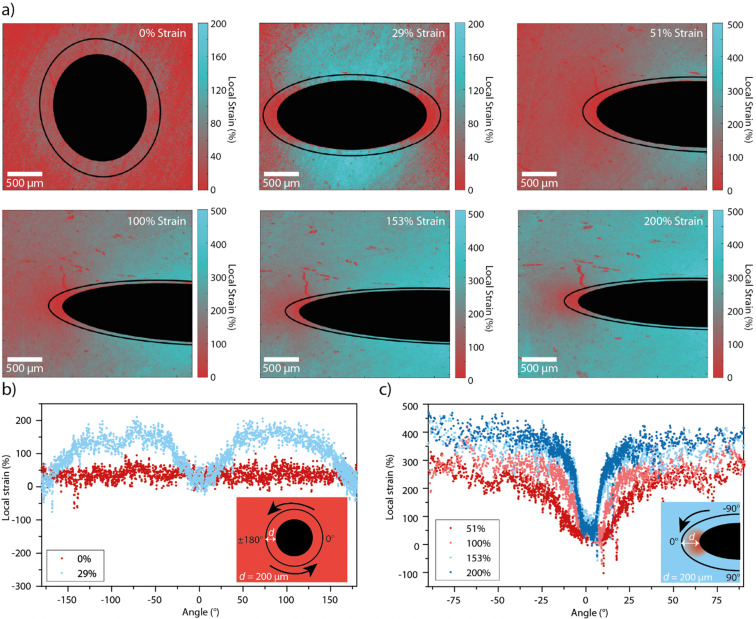
(a) Strain maps of Loop-PU films containing a circular defect (filled black circle or ellipse) in the stress-free (0% applied strain) and stretched (29%, 51%, 100%, 153%, and 200% applied strain) state. The black lines indicate the traces along which the local strain was analysed. (b) and (c) Local strain profiles acquired around the defect along the black circular or elliptical lines in (a) plotted against the polar angle from the center of the hole at an externally applied strain of 0% and 29% (b) and of 51%, 100%, 153%, and 200% (c). Insets indicate the location of the 0°-point in the strain profiles, which run in an anti-clockwise direction around the hole.

Having established the relation between the average mechanochromic response of Loop-PU and the externally applied macroscopic strain, we performed experiments to probe local strains around macroscopic circular defects in the form of 1 mm-wide holes that were cut into the centres of rectangular strips. The films were then subjected to uniaxial tensile deformation (Fig. S5, ESI†), and pairs of fluorescence microscopy images were recorded in the monomer and excimer channels at each strain value. With the calibration data in hand, *I*_M_/*I*_E_ maps of samples containing the macroscopic defects (Fig. S6, ESI†) could be converted into maps displaying the local strain (Fig. 3(a)). This was achieved with a custom MATLAB script that was used to calculate the local strain from the local *I*_M_/*I*_E_ using the calibration curve, for every pixel of the *I*_M_/*I*_E_ map. As for the maps of monomer–excimer ratio in Fig. 2, the strain maps in Fig. 3 are also displayed in pseudo colour. In the absence of external strain, the strain map shows a relatively homogeneous strain distribution around the defect, with the exception of a slight halo that seems to result from uneven illumination, or a small permanent deformation that was induced *via* the creation of the defect (Fig. 3(a)).

At an applied strain of 29%, the strain distribution is markedly more inhomogeneous, as indicated by the blue colour in areas of high local strains on the strain maps. These results are corroborated by strain profiles recorded along a circular or elliptical path around the hole (Fig. 3(b)). These profiles were also created with a MATLAB script, which first defines the hole by tracing a circle (at 0% applied strain) or an ellipse (at higher strain values) on the local strain map, and then extracts the *I*_M_/*I*_E_ values from each pixel along a path that follows the circle or ellipse with the same aspect ratio as the hole and with major vertices 200 μm away from those of the hole. The profile acquired for an external strain of 29% (Fig. 3(b)) shows that local strains of up to *ca.* 150% are reached, notably in areas near the hole that are located around the minor vertices of the defect, while the local strain remains close to 0% in the areas around the major vertices of the defect. When the applied strain is further increased, the contrast in local strain around the defect further increases, though the ratio between the externally applied and the maximal local strain decreases (Fig. 3(a) and (c)). For example, at 200% applied strain, a range of local strains of between 30 and 380% are observed around the defect. While others have observed similar profiles qualitatively,^16,38^ we demonstrate that it is possible to gain quantitative insights over a wide strain range (up to *ca.* 400% local strain) and with an intrinsic approach that is not necessarily limited to imaging surface strains. Moreover, the strain concentration factor, *K*_*ε*_, which is defined as the ratio of the maximum local strain and the externally applied strain, *ε*_max,local_/*ε*_ext_ varies from *ca.* 5 to 2.3 as the externally applied strain increases from 29 to 200%. These figures correspond well to *K*_*ε*_ of 2.5 for a plate containing a hole with 2*r*/*D* = 0.18, where *D* is the width of the sample and 2*r* the diameter of the hole, assuming that the deformation is elastic.^53^ The larger value of *K*_*ε*_ at lower applied strains may result from non-linearity in the stress–strain curve.^54,55^

In order to demonstrate the general applicability of our strain-mapping approach, we applied the methodology to other previously investigated mechanochromic polymers that contain mechano-sensors based on different operating mechanisms and that exhibit different optical responses.^29,49,50^ On account of its versatility and ease of application, we selected a material containing tOPV (Fig. 1(b)), a telechelic poly(ethylene-*co*-butylene) that carries excimer-forming, aggregachromic, cyano-substituted oligo(*p*-phenylene vinylene)s (OPV) at its termini (Fig. 1(b)).^49,50^ Unlike the Loop mechanophore, which must be covalently incorporated into a polymer of interest, tOPV functions as a mechanochromic additive and has been reported to be effective in different polymeric hosts.^49,50^ Here, we used a blend of 0.2 wt% tOPV and a polyurethane (PU) based on methylene diphenyl diisocyanate (MDI) and butane diol (BDO). The application of mechanical force physically separates the cyano-OPV residues at the termini of the tOPV macromolecules, and this causes a ratiometric change in *I*_M_/*I*_E_ similar to that of the Loop mechanophore. We also studied a similar polyurethane into which 0.45 wt% of the rotaxane-based mechanophore Rot was covalently integrated (Fig. 1(c)).^29^Rot is comprised of a dumbbell containing a naphthalene diimide quencher and a cyclic motif bearing a 4-(dicyanomethylene)-2-methyl-6-(4-dimethylaminostyryl)-4*H*-pyran fluorophore. In the idle state, the fluorophore is quenched, but mechanically induced displacement of the fluorophore and quencher causes a pronounced increase in fluorescence intensity. The preparation of tOPV/PU and Rot-PU, their processing into thin films, and the investigation of the mechanoresponses of these materials, including their full spectral characterisation at the macroscopic level, were reported before.^29,49,50^

Calibration curves that correlate the fluorescence microscopy signals of tOPV/PU and Rot-PU films to the externally applied macroscopic strain were established at a strain rate of 50% min^−1^ for tOPV/PU and 345% min^−1^ for Rot-PU. The high strain rate used for the latter materials was applied to minimise effects associated with rapid relaxation processes.^29^ For tOPV/PU, the same process as detailed above for Loop-PU was applied (Fig. S7 and S8, ESI†). Since Rot exhibits a fluorescence turn-on and does not provide a ratiometric signal, only one fluorescence micrograph was recorded at each strain step, and a correction for non-uniform illumination intensity was applied. In order to achieve this, the matrix of fluorescence intensity values obtained from the micrograph was divided element-wise by the array of intensity values obtained at 0% externally applied strain (Fig. S9 and S10, ESI†). The calibrations show that the relation between applied strain and *I*_M_/*I*_E_ is linear for tOPV/PU, as seen for Loop-PU, whereas the fluorescence intensity of Rot-PU increases exponentially with applied strain, which may indicate that the mechano-activation of the rotaxane mechanophore better correlates with the applied stress, rather than strain. A study is presently underway to develop a deeper understanding of the activation of this class of mechanophores. Importantly, however, the existence of different activation mechanisms does not impede the use of the method reported herein for generating mechanochromic strain maps, given that the calibrations between the optical signals and strain are empirically established. We note that, as for Loop-PU, *I*_M_/*I*_E_ is a linear function of the applied strain for **tOPV/PU** when *I*_M_/*I*_E_ is calculated either from fluorescence spectra, as determined in our previous study,^49^ or from fluorescence microscopy images. The non-linear correlation between emission intensity and applied strain for Rot-PU has been observed qualitatively at the macroscopic level.^29^ A quantitative investigation is presently underway.

**Fig. 4:**
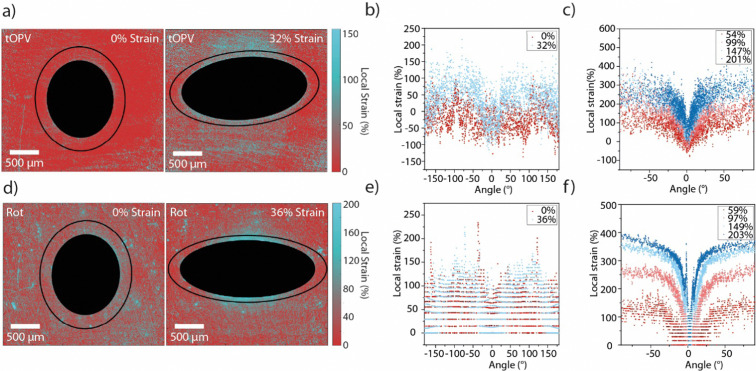
(a) Local strain maps of tOPV/PU films containing circular defects (filled black circle or ellipse), in the unstretched (0% applied strain) and stretched (32% applied strain) states. The black lines indicate the trace along which the local strain profiles around the hole were analysed. (b) Local strain profiles acquired for tOPV/PU at applied strains of 0% and 32%, along the black traces shown in (a) plotted against the angle. (c) Local strain profiles acquired for tOPV/PU at externally applied macroscopic strains of 54%, 99%, 147%, and 201%. (d) Strain maps of Rot-PU films, (containing circular defects filled black circle or ellipse) and are shown in the unstretched (0% applied strain) and stretched (36% applied strain) state. The black lines indicate the trace along which the local strain profiles around the hole were analysed. (e) Local strain profiles acquired for Rot-PU at externally applied macroscopic strains of 0% and 36%, along the black traces shown in (a) plotted against the angle. (f) Local strain profiles acquired for Rot-PU at applied strains of 59%, 97%, 149%, and 203%. Angles are defined in the same manner shown in Fig. 3, with the 0°-point at the major vertex and the profile traced in an anti-clockwise direction.

Films of tOPV/PU and Rot-PU containing circular defects were subjected to uniaxial tensile deformation (Fig. S11 and S12, ESI†). As for the calibration series, pairs of fluorescence microscopy images were recorded in the monomer and excimer channels to generate maps of *I*_M_/*I*_E_ in the case of tOPV/PU (Fig. S13, ESI†) and single fluorescence images to generate intensity maps in the case of Rot-PU (Fig. S14, ESI†). Gratifyingly, the local strain maps from films with a circular defect containing these two different mechanophores largely mirror those acquired with Loop-PU (Fig. 4(a), (d) and Fig. S15, S16, ESI†). At small externally applied strains (32% and 36% respectively for tOPV/PU and Rot-PU), maximum local strains of *ca.* 150% are observed in positions near the hole at an angle of ±90° from the axis of applied tensile strain (Fig. 4(b) and (e)). These results are in excellent agreement with those obtained from Loop-PU, despite the differing mechanical characteristics of the two polyurethanes (Fig. 2(b) and Fig. S8, S11, S12, ESI†). Notably, the strain profiles recorded for tOPV/PU and Rot-PU exhibit more noise than the ones recorded for Loop-PU. In the case of tOPV/PU, this is perhaps related to the fact that tOPV appears to form micrometre-sized aggregates in the PU matrix (Fig. S17, ESI†), which may cause optical inhomogeneities at a microscopic length scale. In the case of Rot-PU, noise may be caused by the low sensitivity of the mechanophore at low strains (Fig. S10, ESI†). Consequently, the local strain profiles become smoother as the applied strain increases (Fig. 4(b) and (e)). At high strains, both data sets show again local strains of up to *ca.* 400%. tOPV/PU reports slightly lower peak local strains of *ca.* 300% (Fig. 4(c)), which could be caused by differences in the mechanical behaviour of the polyurethane matrix. In addition, this mechanophore shows a slight decrease in responsivity, *i.e.*, a decrease in the slope of *I*_M_/*I*_E_*vs.* strain, above 300% applied tensile strain, which could lead to an underestimation of the local strain.^49^

Finally, we demonstrate that this methodology can be applied to map strain distributions around more complex defects at shorter length scales, such as defects produced by inorganic nano- or microparticles. Such fillers are commonly added to polymeric matrices to improve their mechanical properties, such as toughness and stiffness, but they can also cause local stress increases and act as nucleating sites for cracks.^1,6,12,56^ Thus, Loop-PU was compounded with 1 wt% of spherical silica microparticles (diameter = 9–13 μm), and thin films were imaged with a confocal microscope, which permitted the evaluation of optical changes at different depths within the matrix. The samples were imaged *via* excitation with 458 nm light and with filters specific for the transmission of green (*λ*_em_: 508–530 nm) and red (*λ*_em_: 630–735 nm) light, corresponding to the monomer and excimer emission of the PDI residues, respectively. The samples were manually deformed and mounted on glass slides to record 2D confocal images at a depth of 5–6 μm below the surface of the sample (further experimental details can be found in the ESI†).

**Fig. 5:**
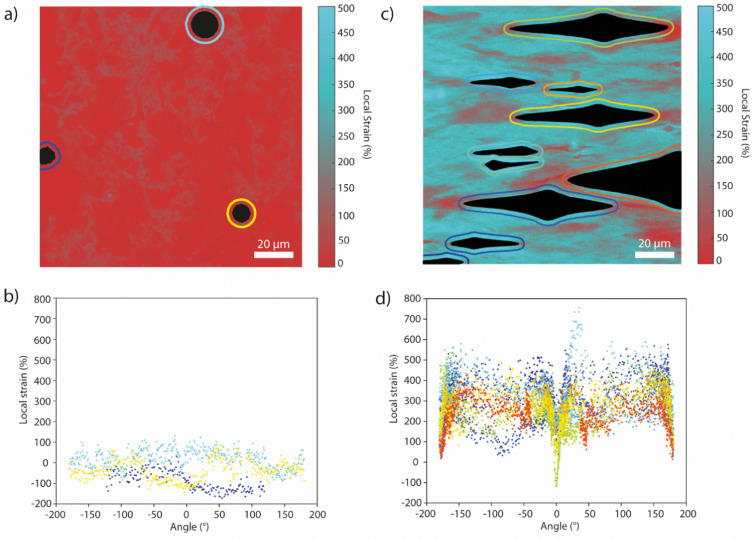
(a) Strain map of a Loop-PU film containing 1 wt% silica beads in the pristine state (0% externally applied strain) and (b) the local strain profiles obtained along the colored lines in (a). (c) Strain map of a film of Loop-PU containing 1 wt% silica beads at 250% uniaxial strain. The colored lines indicate the traces along which the local strains around the holes were analysed. (d) Local strain profiles obtained for Loop-PU at 250% applied strain. The strain profiles are plotted against the polar angle where 0° corresponds to the left major “vertex” of the cavity.

A calibration was again created, using a film of the neat Loop-PU without silica beads (see ESI† for details). Similarly to the calibrations reported above, the sample was progressively stretched from 0 to 200% external strain, stopping at intervals of *ca.* 50% strain to record confocal images. From these micrographs, maps of *I*_M_/*I*_E_ were created in which each pixel corresponds to an area of 0.13 × 0.13 μm (Fig. S18, ESI†); the average value of Δ(*I*_M_/*I*_E_) was then calculated across the entire field of view of the micrograph and plotted against applied strain (Fig. S19, ESI†). Here, the change in *I*_M_/*I*_E_ rather than the absolute value of *I*_M_/*I*_E_ was employed, in order to correct for a small difference in the initial *I*_M_/*I*_E_ between the calibration sample and the sample containing silica beads. At this high magnification, greater inhomogeneity can be observed even in the pristine state without silica beads. Nevertheless, the variations in local strain values around silica spheres in the stress-free state are relatively small (Fig. 5(a) and (b)) and comparable to the variations observed along a line profile through a sample without silica spheres (Fig. S20, ESI†).

The microscopic mechanical response of samples of Loop-PU containing silica beads was then studied upon uniaxial tensile deformation. At 250% applied strain, the PU matrix delaminates from the incompressible silica spheres at the vertices along the axis of deformation, leading to the formation of cavities containing the silica beads (Fig. 5(c)). These cavities are accompanied by marked spatial variations in the spectral characteristics of the surrounding matrix (Fig. 5(d)). Profiles of the local strain at a constant distance of 2.6 μm from the edge of the cavity were obtained with a MATLAB script, in which the cavities were first identified *via* intensity and size thresholding (see ESI† for details). In particular, the matrix polymer adjacent to the delaminated “vertices” exhibits a much lower local strain than the regions closer to the silica spheres, as evidenced by the clear minima that occur at 0 and 180° in the local strain profiles. However, there is a much greater variation observed in the profiles of local strain compared to those obtained around the circular hole defects studied previously. Such variation could be attributed to the size dispersity of the silica beads, interactions between the strain fields around the inclusions and the intrinsic microscopic heterogeneity of the matrix itself, the latter of which is already evident in the pristine state. Interestingly, the local strains in much of the matrix around the defects appear to be significantly in excess of the applied deformation, *i.e.*, *ca.* 500% at an applied strain of 250%. Overall, these microscopic strain maps demonstrate the power in combining supramolecular mechanophores, which offer high strain resolution and sensitivity, with confocal microscopy, which gives access to high spatial resolution in three dimensions. Compared to many of their covalent counterparts,^57^ supramolecular mechanophores exhibit a more sensitive, gradient response to deformation over a wide deformation range, which permits the creation of the highly detailed strain maps.

In conclusion, the results reported herein demonstrate an accessible, versatile method for sensing and mapping spatially varying strain distributions caused by macro- and microscopic defects in polymeric materials. The method combines fluorescence microscopy, tensile testing and image analysis techniques to generate quantitative strain maps around different types of defects with high strain sensitivity and spatial resolution that is limited only by the detection set-up. The three supramolecular mechano-sensing platforms studied, *i.e.*, mechanochromic materials containing the Loop, tOPV, and Rot mechanophores, are sufficiently sensitive to allow the mapping of local strains in elastic polyurethanes over a large strain range. Strain maps acquired using fluorescence microscopy and confocal microscopy reveal that local strains in the vicinity of defects can greatly deviate from the externally applied strain, which emphasises the importance of defects in understanding and predicting the mechanical failure of polymers. We expect that the protocols and analytical methods developed here can be readily applied to other types of mechanophores and polymers, which, depending on the mechanophores employed, could also in principle be applied to monitor local stresses instead of strains. Moreover, the possibility to create strain maps by confocal imaging permits force imaging in three dimensions.

## Data availability

The source data of this study are available from the Zenodo repository at DOI: 10.5281/zenodo.8046613.

## Conflicts of interest

The authors declare no competing interests.

## Supplementary Material

MH-010-D3MH00650F-s001
