# Supplementary material for: Microscopic strain mapping in polymers equipped with non-covalent mechanochromic motifs
Source: Mater Horiz. 2023 Jun 14;10(9):3467–75. doi: 10.1039/d3mh00650f (PMC10463555; doi:10.1039/d3mh00650f)
Supplement: MH-010-D3MH00650F-s001 [file MH-010-D3MH00650F-s001.pdf]

Electronic Supplementary Material (ESI) for Materials Horizons.
This journal is © The Royal Society of Chemistry 2023

Supplementary Information

Microscopic strain mapping in polymers equipped with non-covalent mechanochromic motifs

Hanna Traeger,[a] *Derek Kiebala,*[a] *Céline Calvino,*[b] *Yoshimitsu Sagara,*[c]
Stephen Schrettl,[a,d] *Christoph Weder,* [a] *Jess M. Clough**[,a]

[a] Adolphe Merkle Institute, University of Fribourg,
Chemin des Verdiers 4, 1700 Fribourg, Switzerland

[b] Cluster of Excellence livMatS, University of Freiburg,
Georges-Köhler-Allee 105, D-79110, Freiburg, Germany

[c] Department of Materials Science and Engineering, Tokyo Institute of Technology,
2-12-1, Ookayama, Meguro-ku, Tokyo 152-8552, Japan

[d] Technical University of Munich, TUM School of Life Sciences
Maximus-von-Imhof-Forum 2, 85354 Freising, Germany

* *To whom correspondence should be addressed:*
jessica.clough@unifr.ch

Contents

Supplementary Figures S1–S20 3
Experimental Methods 18
Materials and Instruments 18
Procedures 20
MATLAB Analysis 21
Synthetic procedures 24
NMR Spectra 24
Literature 25

Supplementary Figures S1–S20

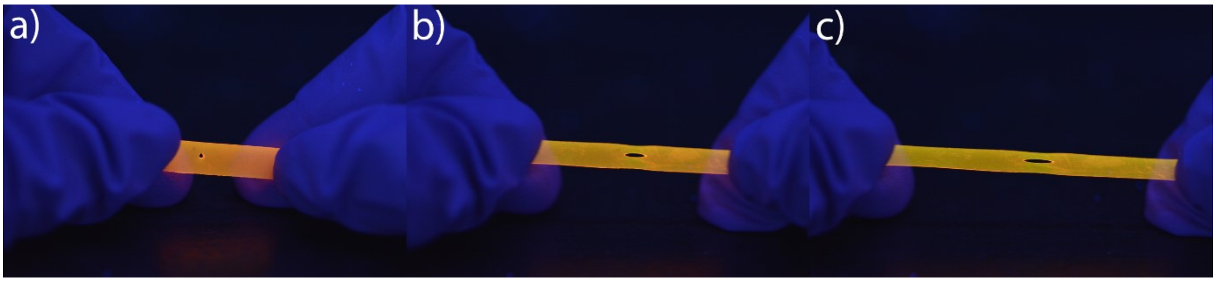

Supporting Figure S1. Photographs under UV light of a film of **Loop-PU** containing a macroscopic circular defect subjected to uniaxial tensile deformation by hand. (a) In the unstrained state, the film emits orange fluorescence emission. (b-c) On applying progressively greater tensile strain, the fluorescence emission in the bulk of the film, away from the defect, turns from orange to green. The colour of the fluorescence emission varies around the defect, with the regions above and below the defect exhibiting greener emission colour than the regions adjacent to major vertices.

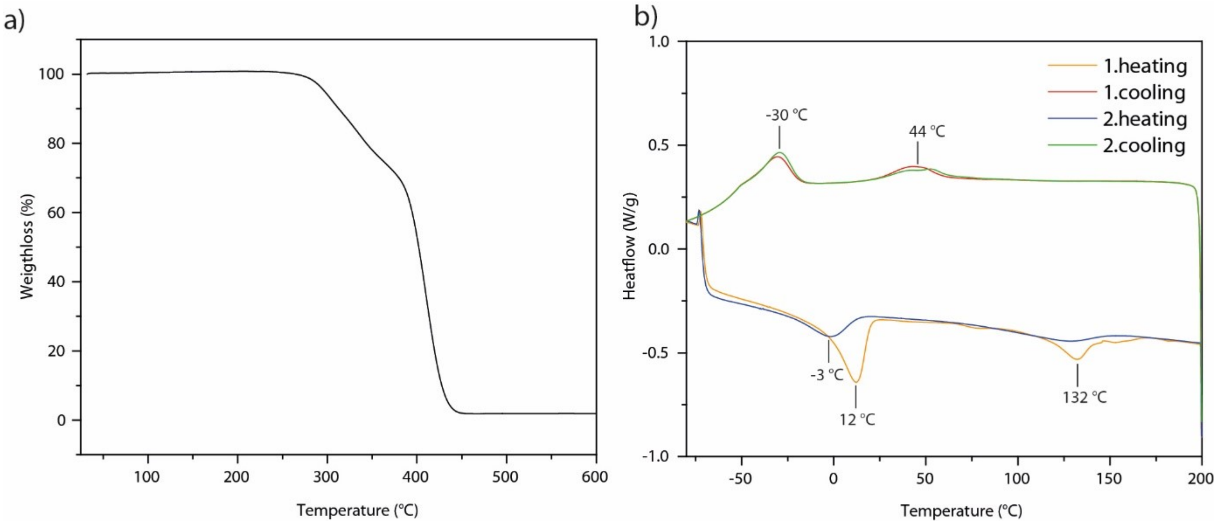

Supporting Figure S2. (a) Thermogravimetric analysis (TGA) of a **Loop-PU** sample recorded with a heating rate of 10°C min^{-1}. Degradation occurred above 230 °C. (b) Differential scanning calorimetry (DSC) trace of **Loop-PU** for two heating and cooling cycles. The trace was recorded with a heating rate of 10°C min^{-1}.

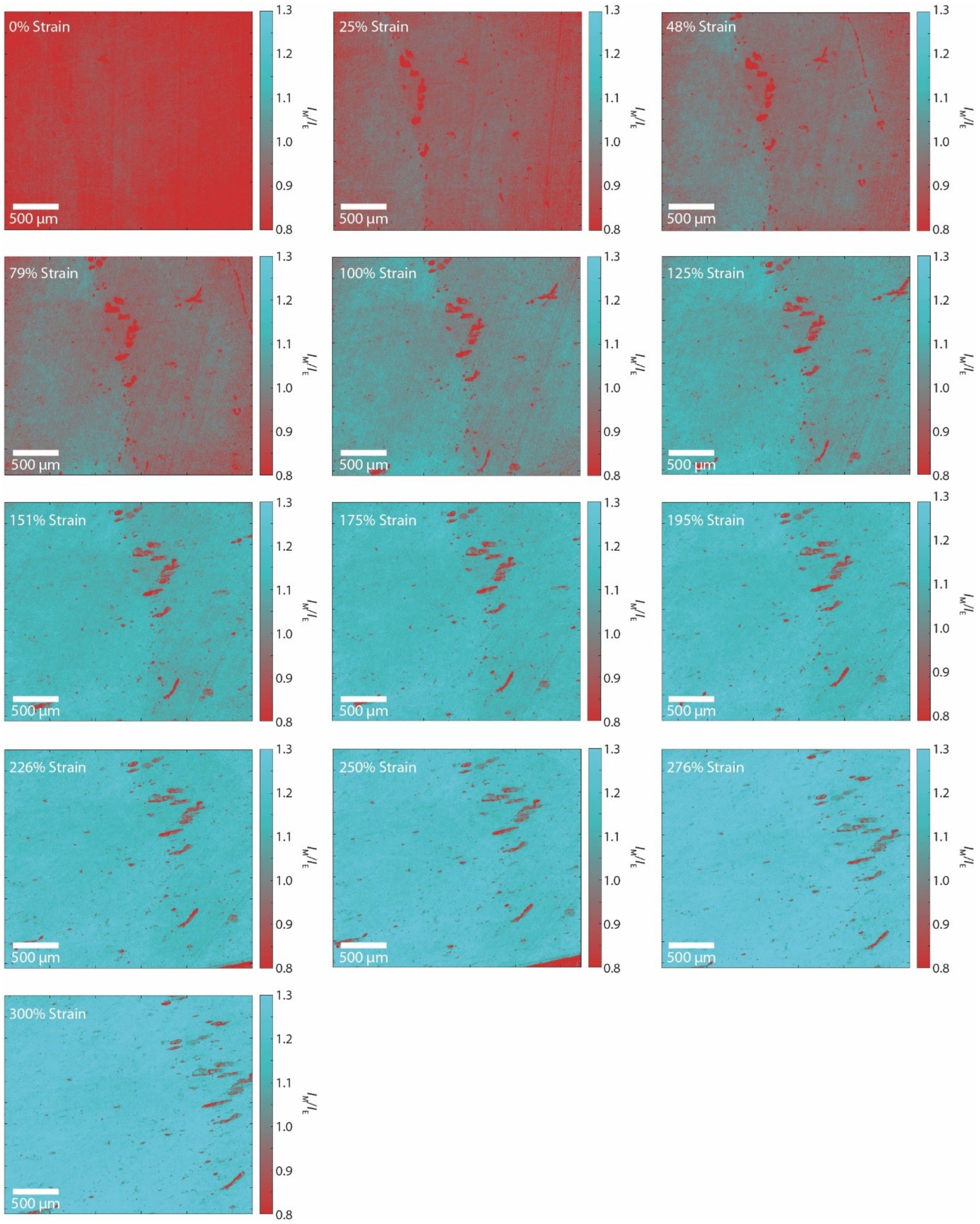

Supporting Figure S3. I_M/I_E maps of **Loop-PU** samples at externally applied strains between 0 and 300%. The samples were stretched at a strain rate of 50% min^{-1} and the application of strain was stopped at intervals of 25% applied strain to record microscopy images with a green (λ_{ex}: 480/40 nm; λ_{em}: 535/50 nm) and a red filter (λ_{ex}: 469/35 nm; λ_{em}: 620/52 nm). Details of the procedure used to obtain the I_M/I_E maps are given in the MATLAB Analysis section (*vide infra*).

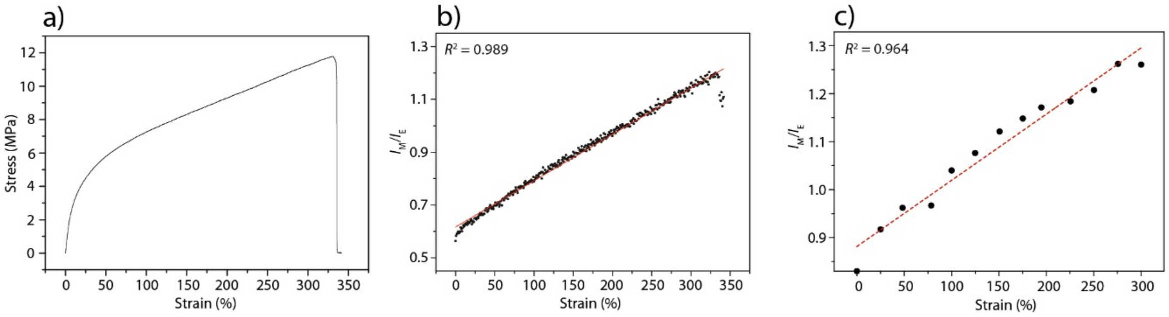

Supporting Figure S4. Comparison of correlations between the applied uniaxial tensile strain and (a) the stress, (b) the monomer-to-excimer emission intensity ratio, I_M/I_E and determined by *in-situ* fluorescence spectroscopy, and (c) I_M/I_E determined by *in-situ* fluorescence microscopy. Data shown in (a) and (c) were acquired at a strain rate 50% min^{-1} and were taken from reference 1. Data shown in (c) were based on the fluorescence microscopy images recorded through green and red filters shown in **Figure S3**, following the analysis protocol described in the main manuscript. Note that the fact that the slopes of the linear fits through the data shown in (b) and (c) (0.0013 and 0.0018, respectively) are nearly identical is serendipitous, as these functions depend on the specific acquisition parameters, such as the specific wavelengths and -ranges at which I_M and I_E are determined. Nevertheless, the fact that I_M/I_E and strain are linearly correlated in both cases reflects that macroscopic spectroscopic measurements can be substituted by imaging techniques that additionally provide spatial resolution.

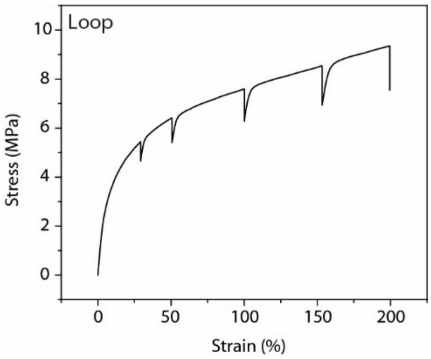

Supporting Figure S5. Stress-strain curves samples of **Loop-PU** containing a macroscopic circular hole**.** Samples of **Loop-PU** were stretched with a strain rate of 50% min^{-1}. The measurements were stopped at ~29 %, 51 %, 100 %, 153%, and 200 % applied strain to record microscopy images.

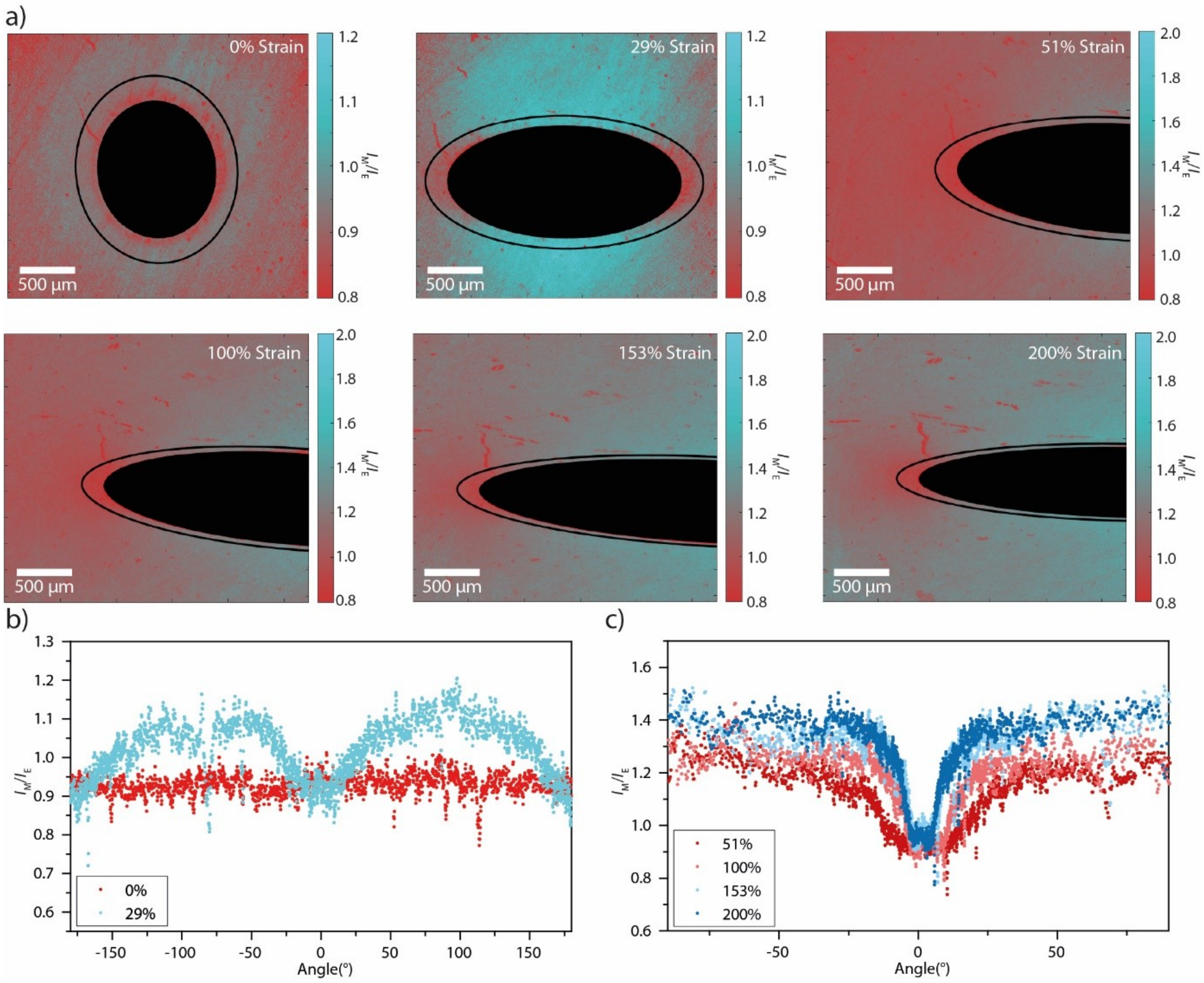

Supporting Figure S6. I_M/I_E maps (a) of films of **Loop-PU**, calculated from microscopy images recorded with a green (λ_{ex}: 480/40 nm; λ_{em}: 535/50 nm) and a red filter (λ_{ex}: 469/35 nm; λ_{em}: 620/52 nm). The sample was inflicted with a hole (filled black circle or ellipse) and is shown in the unstretched (0% applied strain) and stretched (ca. 29%, 51%, 100%, 153%, and 200% applied strain) state. The black lines indicates the trace along which the I_M/I_E around the hole were analysed. I_M/I_E profiles acquired for **Loop-PU** at an externally applied strain of (b) 0% and 29% and (c) 51%, 100%, 153%, and 200% along the black traces shown in (a) are plotted against the polar angle from the center of the hole.

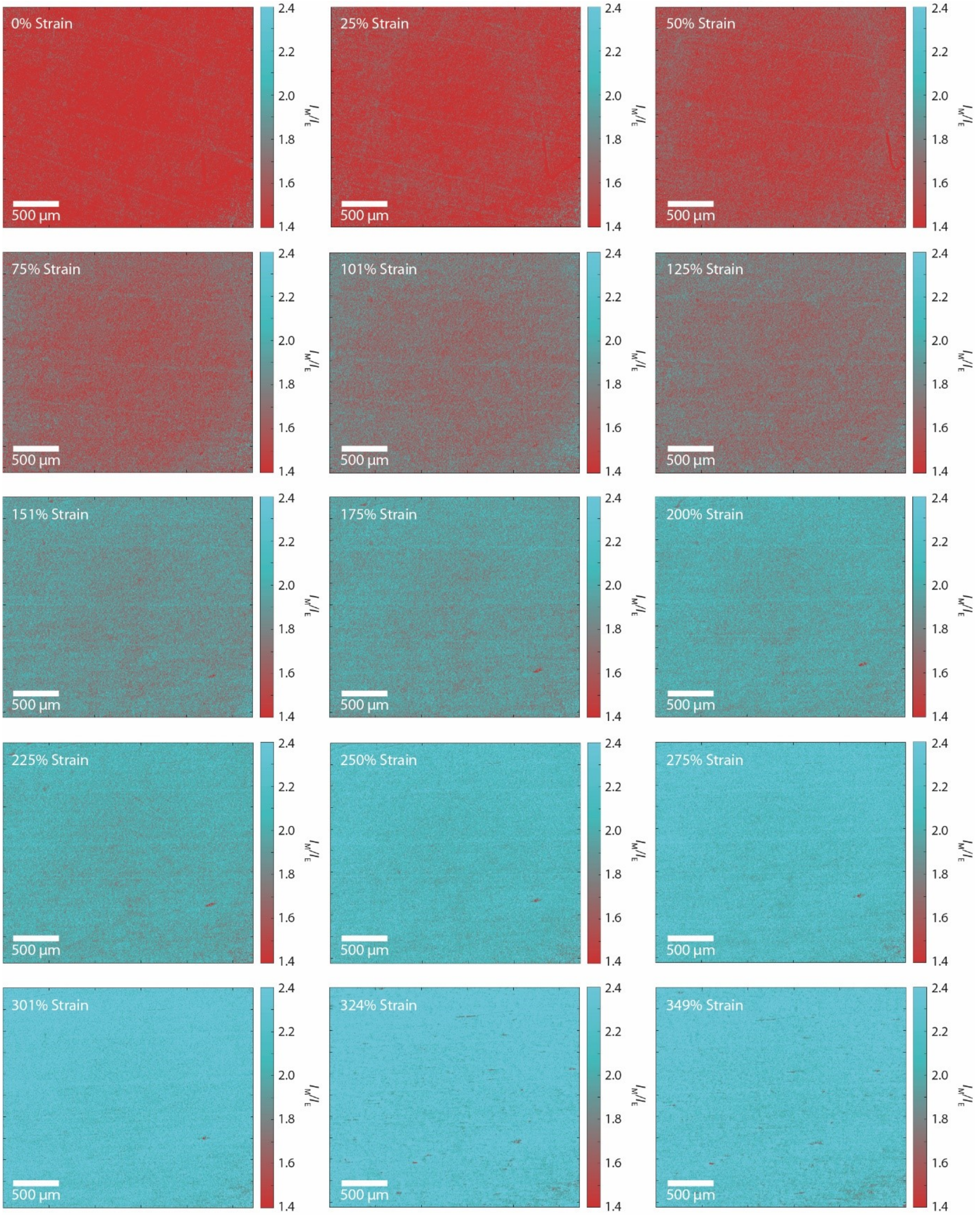

Supporting Figure S7. I_M/I_E maps of **tOPV/PU** samples at applied strains between 0% and 349%. The samples were stretched at a strain rate of 50% min^{-1} and the application of strain was stopped at intervals of 25% applied strain to record microscopy images with a green (λ_{ex}: 480/40 nm; λ_{em}:535/50 nm) and a red filter (λ_{ex}: 469/35 nm; λ_{em}: 620/52 nm). Details of the procedure used to obtain the I_M/I_E maps are given in the MATLAB Analysis section (*vide infra*).

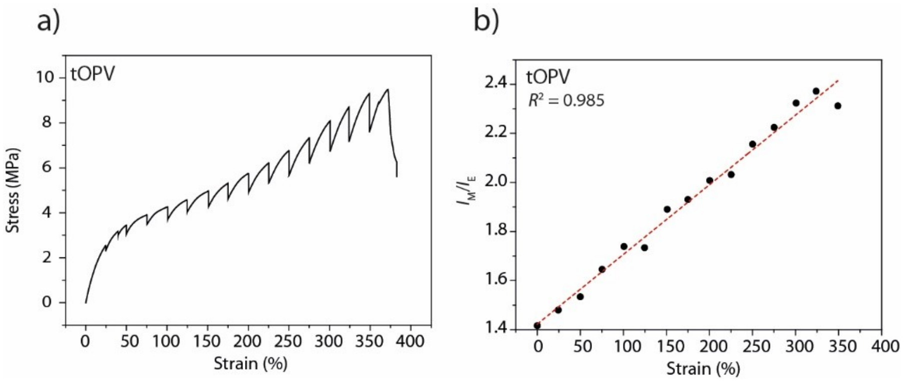

Supporting Figure S8. (a) Stress-strain curve for a sample of **tOPV/PU.** The sample of **tOPV/PU** was stretched with a strain rate of 50% min^{-1}. The measurement was stopped at intervals of 25% applied strain to record microscopy images and plot calibration curves (b) correlating I_M/I_E to externally applied macroscopic strain.

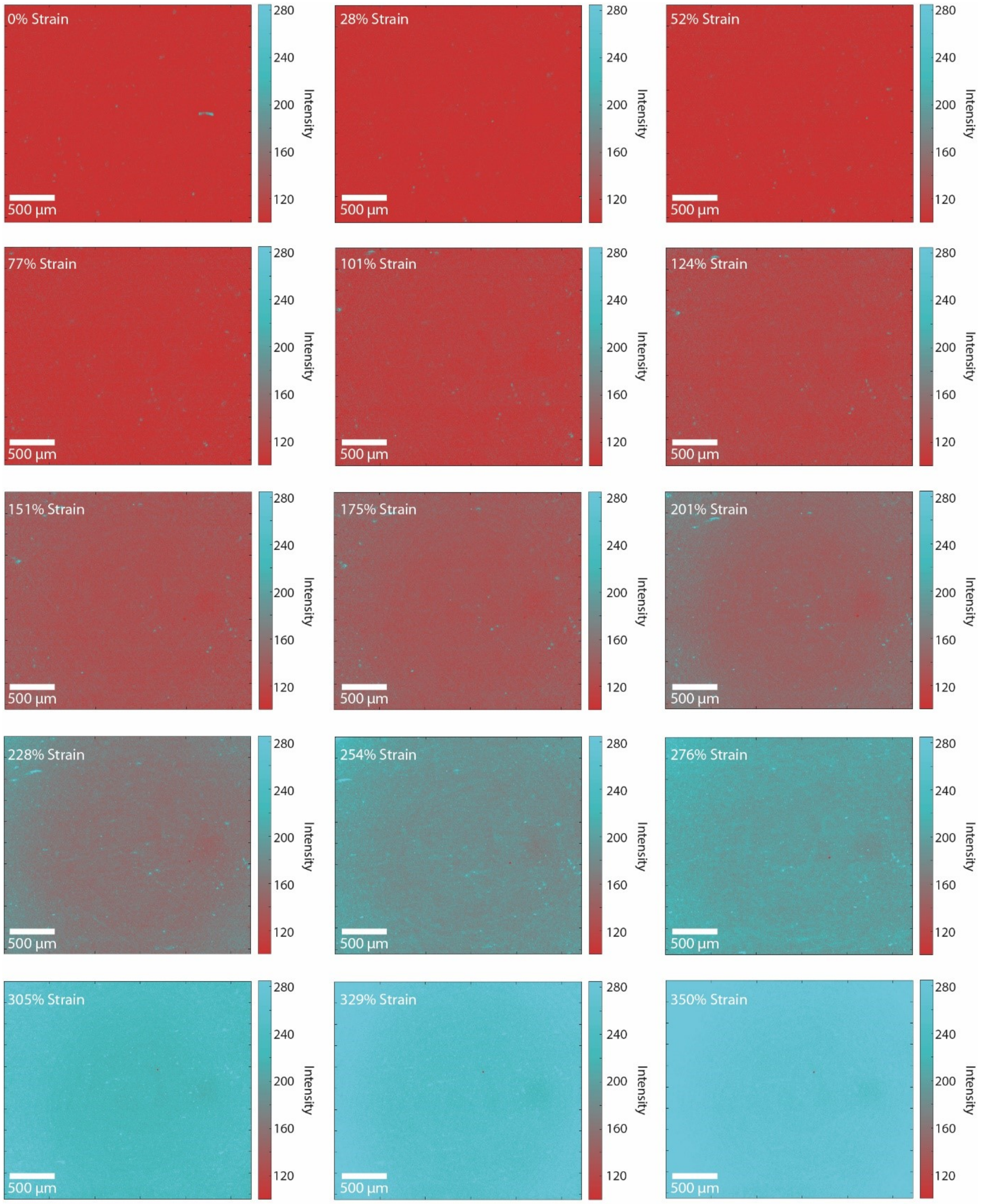

Supporting Figure S9. Fluorescence intensity maps of **Rot-PU** samples stretched to applied strains between 0% and 350%. The sample was stretched at a strain rate of 1000 μm/s and the application of strain was stopped at intervals of 25% applied strain to record microscopy images under UV excitation (λ_{ex} = 330-385 nm; λ_{em} = 420-900 nm). Details of the procedure used to obtain the intensity maps are given in the MATLAB Analysis section (*vide infra*).

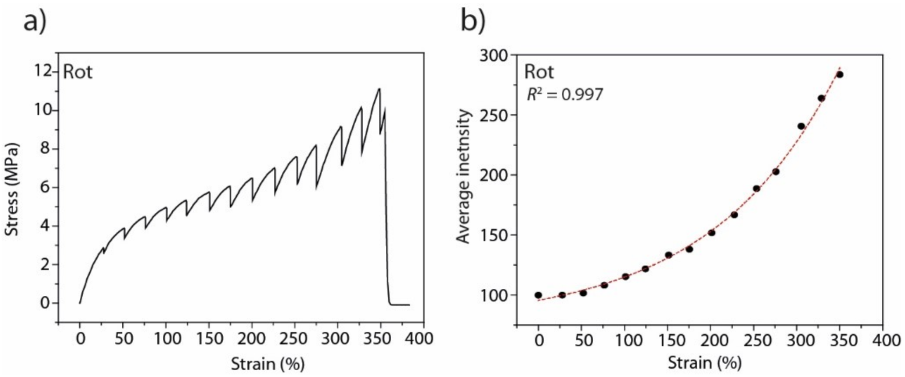

Supporting Figure S10. Stress-strain curve for a sample of (a) **Rot-PU.** The samples of **Rot-PU** were stretched with a strain rate of 50% min^{-1}. The measurement was stopped at intervals of 25% applied strain to record microscopy images and plot calibration curves (b) correlating average intensity to externally applied macroscopic strain.

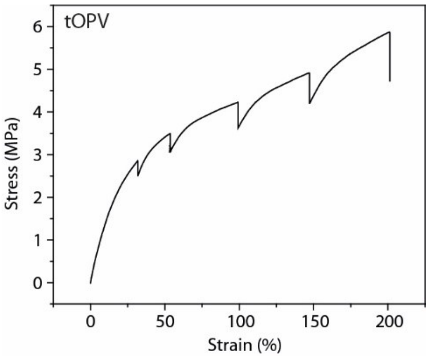

Supporting Figure S11. Stress-strain curve of a sample of **tOPV/PU** containing a macroscopic circular hole**.** The sample of **tOPV/PU** was stretched with a strain rate of 50% min^{-1}. The measurement was stopped at 32 %, 54 %, 99 %, 147%, and 201 % applied strain to record microscopy images.

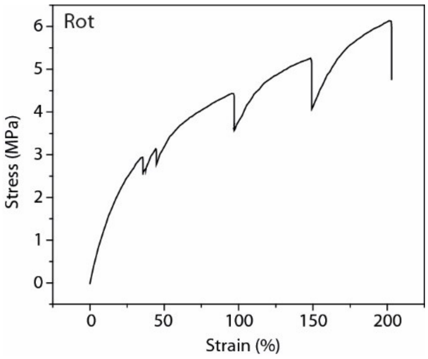

Supporting Figure S12. Stress-strain curve of **Rot-PU** containing a macroscopic circular hole**.** The sample of **Rot-PU** was stretched with a strain rate of 50% min^{-1}. The measurement was stopped at 32%, 45%, 97%, 149%, and 203% applied strain to record microscopy images.

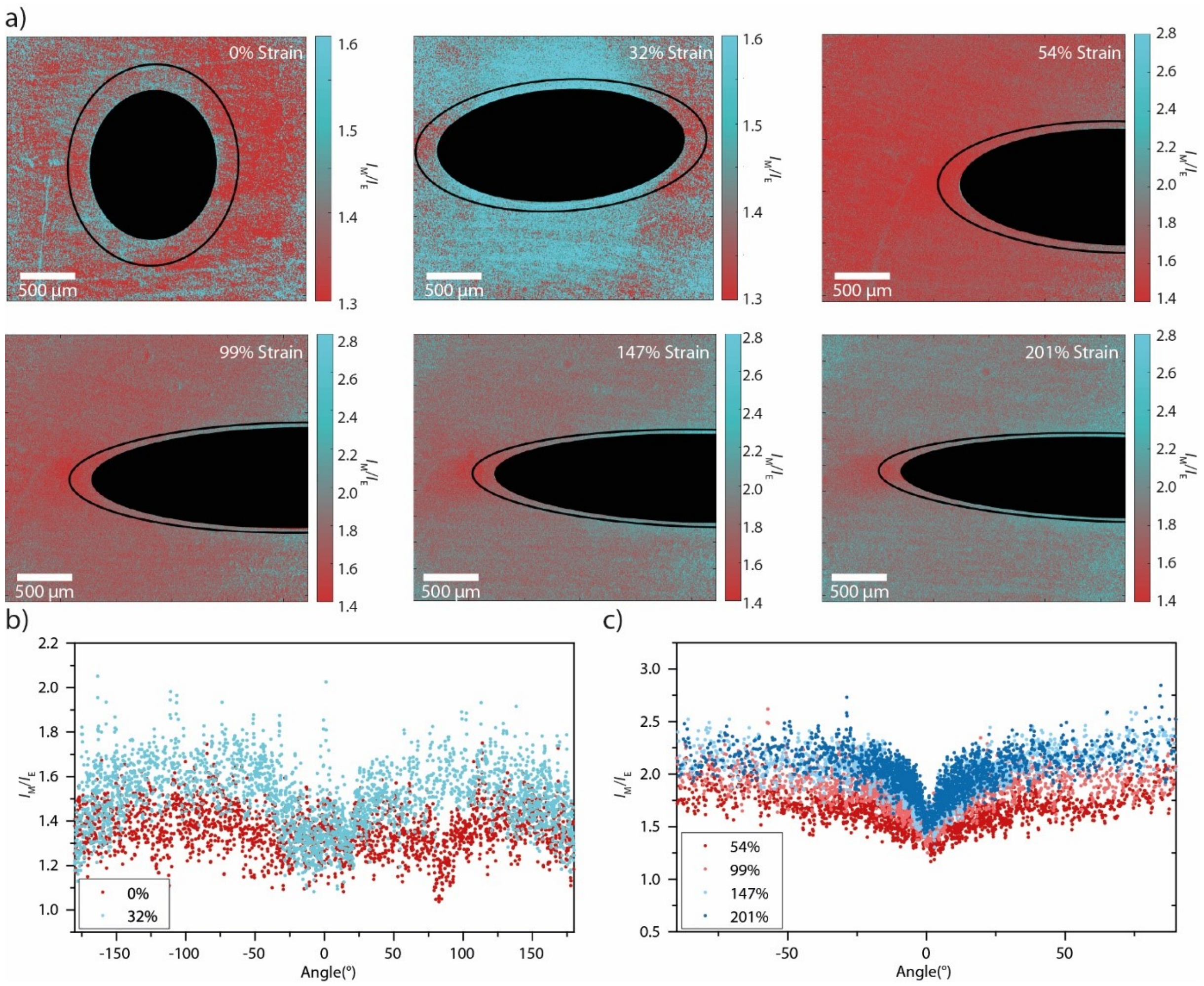

Supporting Figure S13. I_M/I_E maps (a) of films of **tOPV/PU**, calculated from microscopy images recorded with a green (λ_{ex}: 480/40 nm; λ_{em}: 535/50 nm) and a red filter (λ_{ex}: 469/35 nm; λ_{em}: 620/52 nm). The sample was inflicted with a hole (filled black circle or ellipse) and subjected to applied strains of 0%, 32%, 54%, 99%, 147%, and 201%. The black lines indicate the trace along which I_M/I_E around the hole was analysed. I_M/I_E profiles acquired for **tOPV/PU** at 0% and 32% (b) and 54%, 99%, 147%, and 201% applied strain along the black traces shown in (a) plotted against the polar angle from the center of the hole. Details of the procedure used to obtain the I_M/I_E maps are given in the MATLAB Analysis section (*vide infra*).

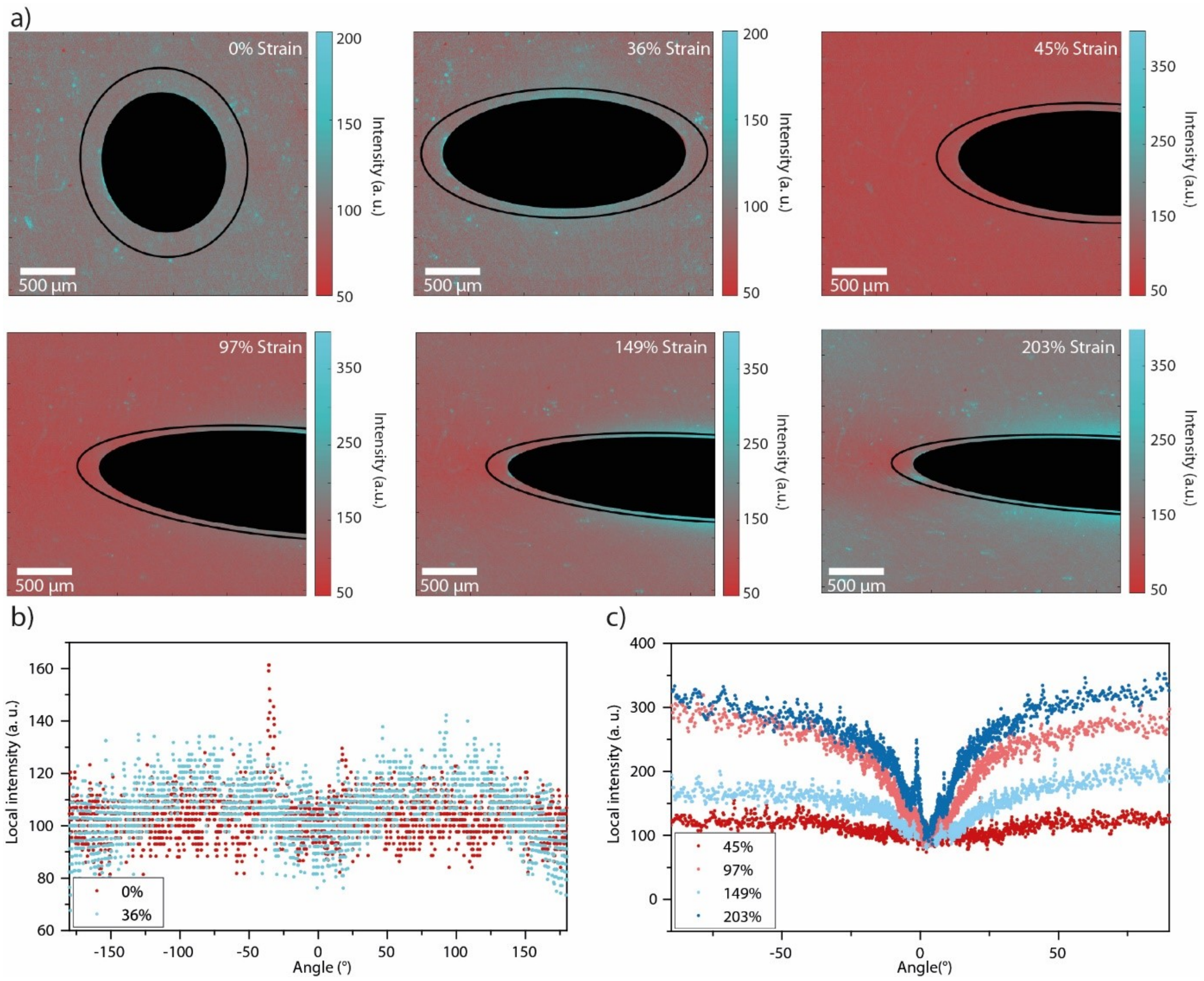

Supporting Figure S14. (a) Fluorescence intensity maps of films of **Rot-PU**. The sample was inflicted with a hole (filled black circle or ellipse) and is shown in the unstretched (0% applied strain) and stretched (ca. 36%, 45%, 97%, 149%, and 203% applied strain) state. The black lines indicate the traces along which the local fluorescence intensity around the hole were analysed. Local fluorescence intensity profiles acquired for **Rot-PU** at 0% and 32% (b) and 54%, 99%, 147%, and 201% applied strain along the black traces shown in (c) plotted against the polar angle from the center of the hole.

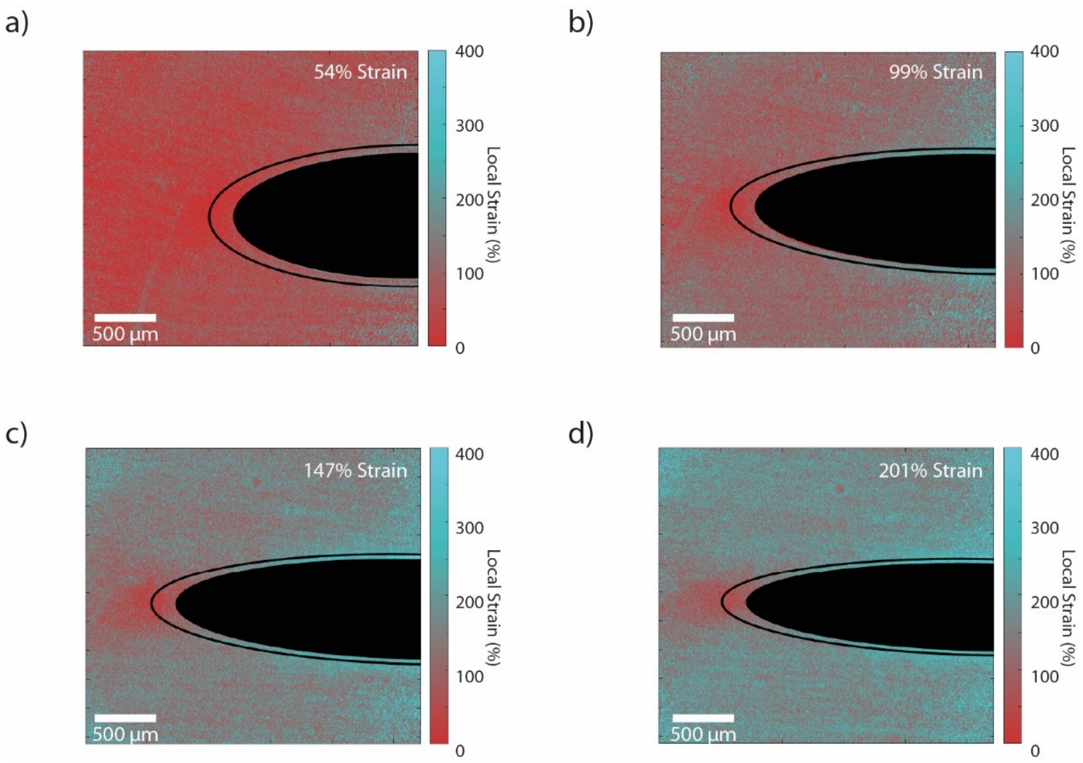

Supporting Figure S15. Local strain maps of films of **tOPV/PU**, calculated from microscopy images recorded with a green (λ_{ex}: 480/40 nm; λ_{em}: 535/50 nm) and a red filter (λ_{ex}: 469/35 nm; λ_{em}: 620/52 nm). The samples were inflicted with a hole (filled black circle or ellipse) and are shown at 54%, 99%, 147%, and 201% applied strain. The black lines indicate the trace along which the I_M/I_E around the hole was analysed.

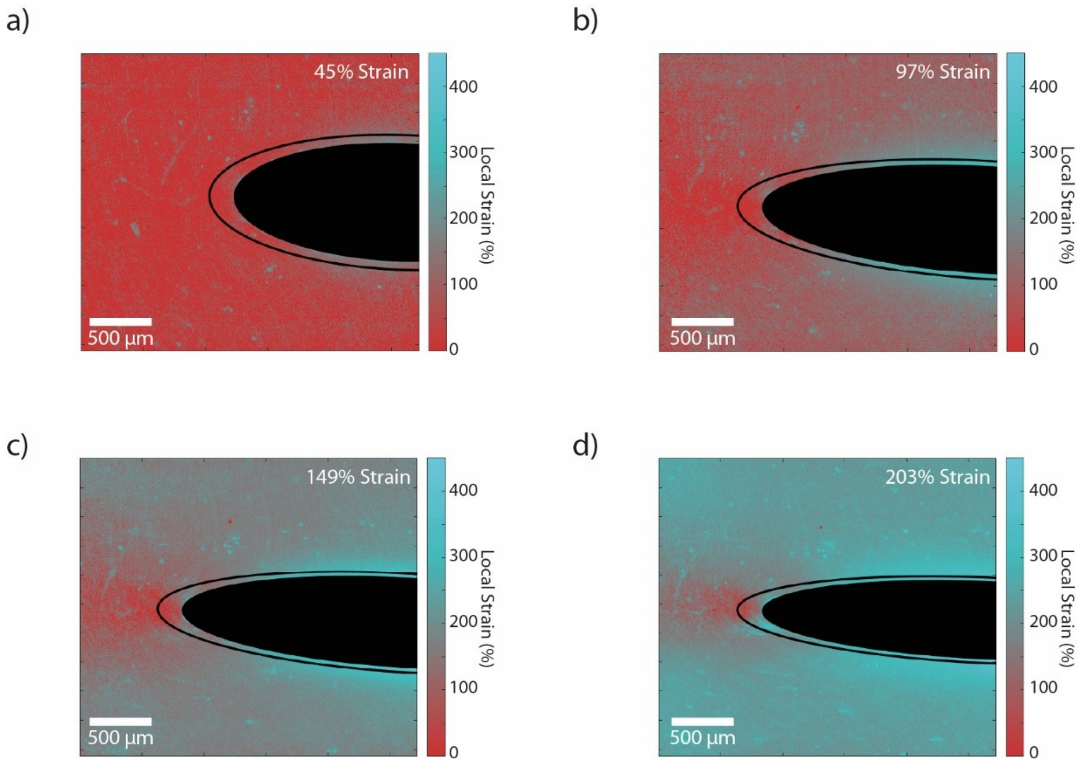

Supporting Figure S16. Local strain maps of films of **Rot-PU**. The samples were inflicted with a hole (filled black circle or ellipse) and are shown at ca. 45%, 97%, 149%, and 203% applied strain. The black lines indicate the traces along which the local fluorescence intensity around the hole was analysed.

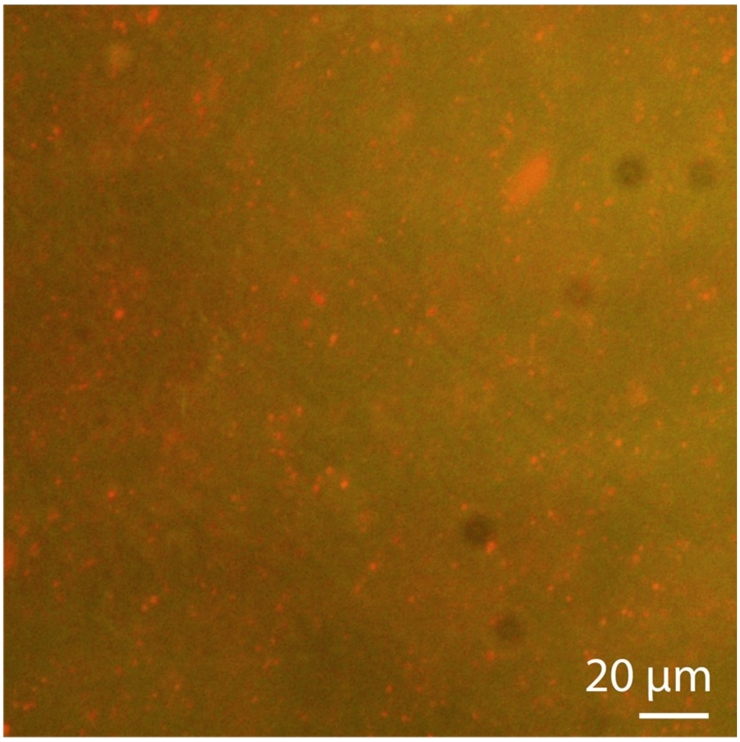

Supporting Figure S17. Widefield fluorescence micrograph of tOPV/PU, recorded under UV excitation (λ_{ex} = 330-385 nm; λ_{em} = 420-900 nm).

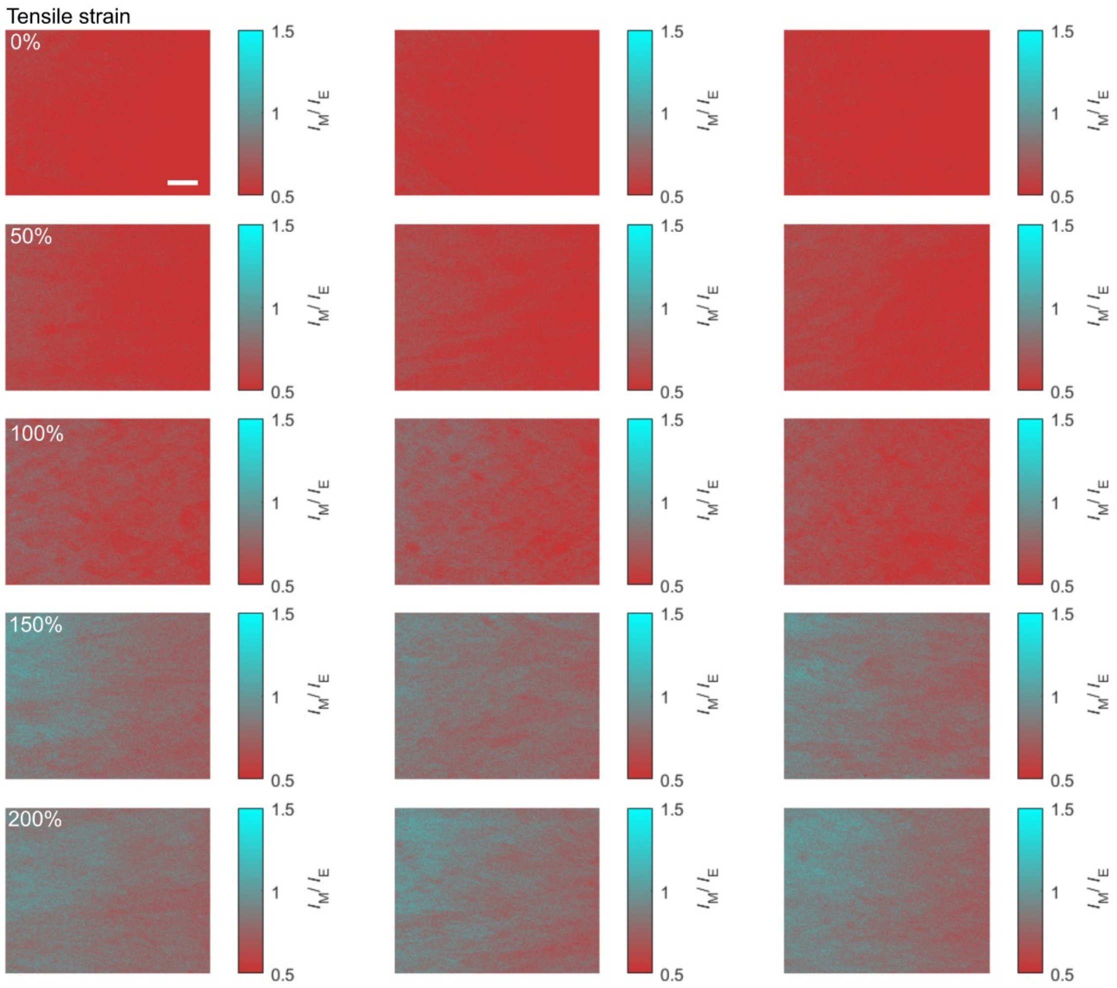

Supporting Figure S18. Maps of the monomer-to-excimer emission intensity ratio, I_M/I_E, for **Loop-PU** at five different applied uniaxial tensile strains between 0 and 200%, calculated from confocal micrographs with a MATLAB script. Micrographs were obtained using laser excitation at 458 nm, and the emission was recorded in two channels: 508-530 nm (monomer, false-color blue) and 630-735 nm (excimer, false-color red). Details of the procedure used to obtain the I_M/I_E maps are given in the MATLAB Analysis section (*vide infra*). For tensile testing, the sample was stretched by hand and clamped in place on a microscope slide. For each applied strain, three different spots of the same sample were imaged with a red and green filter (*vide supra*).

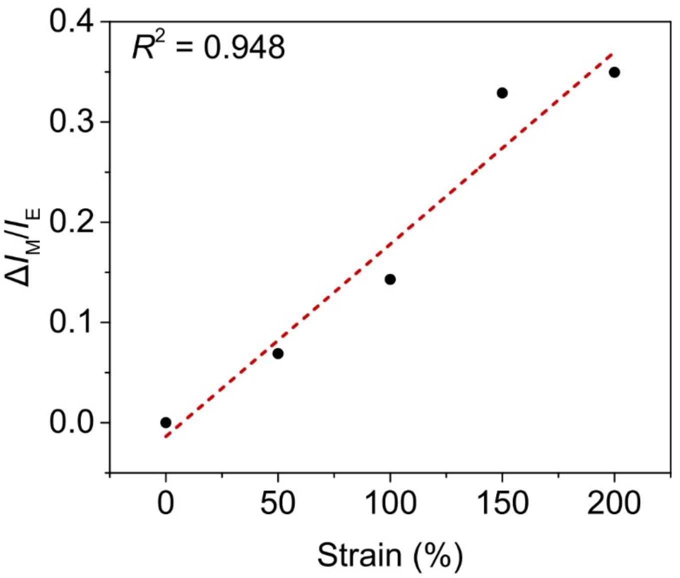

Supporting Figure S19. Calculated average of monomer-to-excimer emission intensity ratios, I_M/I_E, for **Loop-PU** from the images in Figure S15 vs. externally applied macroscopic strain.

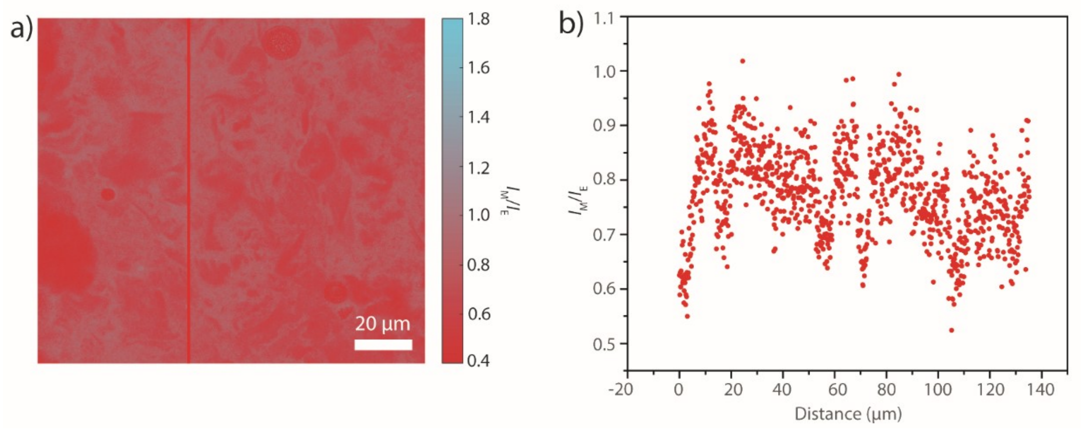

Supporting Figure S20. (a) Map of the monomer-to-excimer emission intensity ratio, I_M/I_E, for **Loop-PU** containing 1 wt% silica beads in the pristine state at 0% applied strain. (b) Profile of I_M/I_E taken along red line in (a), revealing local heterogeneities present in the polymer matrix before a strain is applied.

Experimental Methods

Materials and Instruments

Materials. Poly(tetrahydrofuran) (2000 g/mol), dibutyltin dilaurate, hexamethylene diisocyanate, 4,4′-methylenebis(cyclohexyl isocyanate), 1,4-butanediol and glass beads (diameter 9-13 µm) were purchased from Sigma Aldrich. All solvents were purchased from ACROS, Fisherbrand, or Reactolab. 1,4-Butane diol was distilled once prior to use and poly(tetrahydrofuran) was dried at 60 °C for two days on high vacuum and stored in a glove box. All reactions were performed under Schlenk conditions with oven dried glassware. Deuterated solvents were bought from Cambridge Isotope Laboratories, Inc.

Nuclear magnetic resonance (NMR) spectroscopy. NMR spectra were measured at 297.2 K on a Bruker Avance DPX 400 spectrometer at frequencies of 400.19 MHz for 1H nuclei and 100.63 MHz for 13C nuclei. Residual solvent peak of $CDCl_3$ were set as a reference. Spectra were analysed with MestReNova software suite (v 12.0) to obtain chemical shifts (δ), reported in parts per million (ppm), and coupling constants in Hz (multiplicity: s = singlet, d = doublet, dd = double doublet, t = triplet, m = multiplet, br = broad signal).

Fluorescence microscopy. Microscopy images were acquired at 5x magnification using an Olympus BX51 microscope bearing an Olympus DP72 high-resolution camera. The samples were imaged in reflectance mode using an X-Cite Series 120-Q Mercury vapor short arc lamp as the excitation source (λ_{ex} = 330–385 nm). The white balance was adjusted with a standard white diffuser. For Loop-PU and tOPV/PU, images were recorded with a green filter (λ_{ex}: 480/40 nm; λ_{em}: 535/50 nm, Olympus U-MF2) and with a red filter (λ_{ex}: 469/35 nm, Thorlabs MF469-35; λ_{em}: 620/52 nm, Thorlabs MF620-52), where the numbers indicate the wavelength of peak transmission and the full width at half maximum, respectively. For Rot-PU, single images were recorded with a wideband UV filter (λ_{ex} = 330-385 nm; λ_{em} = 420-900 nm, Olympus U-MWU2).

Microtensile tester. Rectangular compression molded or solvent-cast films (40 × 5.5 × 0.05 mm length × width × thickness) were subjected to uniaxial tensile deformation at a strain rate of 50% min^{-1} or 1000 um/s using a Linkam TST350 microtensile stage equipped with a 20 N load cell. Data were recorded with Linksys32 software. For *in-situ* fluorescence microscopy measurements, the translation stage of the microscope was removed and the microtensile tester was clamped in place below the microscope objectives.

Thermogravimetric analysis (TGA). TGA measurements were conducted in the temperature range of 25 to 600°C and with a heating rate of 10 °C min^{-1} on a Mettler Toledo TGA/DSC 1 STAR system.

Differential scanning calorimetry (DSC). DSC measurements comprised of two heating and cooling cycles were performed under a nitrogen atmosphere with heating and cooling rates of 10 °C min^{-1} over a temperature range of –80 to 200 °C on a Mettler Toledo DSC 2 STAR system. The melting temperature, T_m is reported as the temperature at which the minimum of the melting peak occurs, and

the crystallisation temperature, T_c is reported as the temperature at which the maximum of the crystallisation peak occurs.

Confocal microscopy imaging. Confocal microscopy images were acquired on a Zeiss LSM 710 meta confocal laser scanning microscope (CLSM) equipped with an Ar laser (max. power 25 mW) and a spectrometer allowing acquisition of emitted light in two channels at nanometer precision within the visible range. All data was recorded using a 63X/1.3NA oil lens at a lateral resolution of 0.132 μm. For further details, see Procedures section.

Procedures

Sample preparation for microscopy.

Loop-PU films were compression molded at 150 °C for 3 min at 4 tons and slowly cooled to room temperature. The as-prepared samples were cut into films with dimensions of 40 × 5.5 × 0.2 mm (length × width × thickness). A hole was cut into the film using a 1 mm disposable biopsy punch with plunger (Integra Miltex). Pristine samples, not containing a hole, were used for the calibration curve.

tOPV was synthesised as previously reported.[2] Blends with Texin 985 (Covestro; M_n = 111 kDa, Đ = 2.0) and **tOPV** were prepared as previously reported.[3] Films with a concentration of 0.2 wt% tOPV were cut into strips with dimensions of 40 × 5.5 × 0.2 mm (length × width × thickness). A hole was cut into the film with a 1 mm disposable biopsy punch with plunger (Integra Miltex). Pristine samples, not containing a hole, were used for the calibration curve.

Rot and **Rot-PU** were synthesised and films were prepared as previously reported.[4] A hole was cut into the film with a 1 mm disposable biopsy punch with plunger (Integra Miltex). Pristine samples, not containing a hole, were used for the calibration curve.

Measurement conditions for widefield fluorescence microscopy.

Loop-PU and tOPV/PU. Fluorescence microscopy images were recorded with a 5x air objective and the exposure times were kept constant throughout the experiment. One image was recorded with a green filter (λ_{ex}: 480/40 nm; λ_{em}: 535/50 nm) and a second image with a red filter (λ_{ex}: 469/35 nm; λ_{em}: 620/52 nm). A MATLAB script was used to divide the arrays of grayscale intensities corresponding to the green and red fluorescence images element-wise to produce an I_M/I_E map, and to analyse I_M/I_E along a line around the defect (for further details, see MATLAB Analysis section). The same procedure was repeated on pairs of images taken at various externally applied strains that were stretched with a strain rate of 50 % min^{-1} between the strain-points.

Rot-PU. Fluorescence microscopy images were recorded with a 5x air objective and the exposure times were kept constant throughout the experiment. One image was recorded with a UV filter. In order to correct for non-uniform fluorescence intensity, a MATLAB script was used to divide the array of grayscale intensities corresponding to the recorded image by a second array corresponding to the fluorescence intensity recorded from a pristine sample at 0% applied strain. As for Loop-PU and tOPV/PU, a MATLAB script was also used to define the hole and subsequently calculate the intensity along a line around the notch in a spatially resolved manner (for further details, see MATLAB Analysis section). The same procedure was repeated on pairs of images taken at various externally applied strains that were stretched with a strain rate of 345 % min^{-1} (1000 µm s^{-1}) between the strain-points.

Measurement conditions for confocal microscopy.

Channel 1 was recorded using laser excitation at 458 nm and emission was recorded between 508 and 530 nm. Channel 2 was recorded using laser excitation at 458 nm and emission was recorded between 630 and 735 nm. The recorded emission ranges for channels 1 and 2 correspond to the emission of

perylene diimide predominantly in its monomeric and excimeric states, respectively. A laser attenuation transmission of 9%, pinhole diameter of 0.7 μm (equivalent to 1 Airy unit), master gain of 700, digital offset of 0, and digital gain of 1.2 were used to acquire all images using the Zeiss Zen 2010 software unless otherwise noted. Two-dimensional (2D) CLSM images were acquired at a resolution of 1024 × 1024 pixels and 16-bit depth with dimensions of 134.8 × 134.8 μm, pixel size of 0.13 μm, pixel dwell of 100.85 μs and scan time of 1min 54 sec per image. Images were recorded at a depth, *d* of 5-6 μm in the sample, where d = 0 μm is the surface of the sample in contact with the coverslip, and the thickness of the confocal cross-section was 0.7 μm. For each sample, two-dimensional images were recorded at 1–3 spots and analysed.

MATLAB Analysis

The micrographs obtained from the fluorescence microscope are 1360 × 1024 .tif files in a 16-bit RGB format. For the results with **Loop** and **tOPV**, each strain map is obtained from two micrographs, one recorded with the green filter and the other with the red filter. These are first read into MATLAB (imread), converted to grayscale (rgb2gray), then to a numerical array with double precision which is necessary for the following numerical manipulation (im2double). The array corresponding to the green fluorescence micrograph is divided element-wise by the array corresponding to red fluorescence micrograph (bsxfun). The I_M/I_E ratios obtained in each pixel of this array are then used to calculate local strain values, using the parameters obtained from the calibration. For both the loop and tOPV results, these parameters are the slope and intercept of a straight line fit to I_M/I_E vs. externally applied strain, obtained from calibration experiments as described in the main article (*i.e.,* $\varepsilon_{local} = (I_M/I_E - intercept_{calib})/slope_{calib}$). A first local strain map is then visualised for inspection (imagesc). The x- and y-axes of this plot can be scaled to show the physical dimensions of the micrograph (rather than the size of the image in pixels), using the scaling factor in μm/ pixel from the scale-bar in the microscope images. The hole is defined by drawing an ellipse manually on the strain map (drawellipse). The elliptical path along which a local strain profile is extracted is defined geometrically as having the same aspect ratio as the ellipse corresponding to the hole, with its major vertices fixed at a distance corresponding to 200 μm away from those of the hole. In order to generate the profile, a mask is first applied to the image, in which all the pixels contained within the elliptical path are defined as '1', and those outside as '0' (createMask). The (x,y)-coordinates of the pixels on the boundary between the two regions are then extracted (bwboundaries). These coordinates are then used to obtain the values of the specific pixels in the local strain map. The (x,y)-coordinates are converted to vectors from the center point of the hole by subtracting the coordinates of the center point, then to polar coordinates (cart2pol). The values of the angle θ, which is angle between the line from the center of the ellipse to the pixel and the line from the center of the ellipse to the major vertex, are sorted from $-\pi$ to π (sort). In the case of externally applied strains less than 50%, an angle of 0° is obtained from the line drawn from the center to the right-hand major vertex; at applied strains greater than 50%, only one major vertex is visible in the micrograph,

and the 0° point is defined there. The profile is traced in a counter-clockwise direction. The θ-values are paired with the corresponding local strain values and the local strain vs. θ profiles can be plotted. At externally applied tensile strains greater than 50%, the hole became too large to be recorded entirely within a single micrograph. In these cases, the same analytical approach is employed, but with the additional step of extending the strain map with an area of 1300 × 1024 pixels that have a value of '0' (padarray). The hole can then be defined and the local strain profile extracted as above (the strain profile contains 0-values for the part of the sample not shown in the micrographs, but this part is removed). To generate the strain maps showing the elliptical paths (*e.g.,* Figure 3a), the "padding" pixels are removed.

For results with **Rot**, local strain maps are generated from single micrographs of mechanically activated fluorescence intensity. The images are read in, converted to grayscale and converted to double precision arrays, as before. They are then corrected for uneven illumination intensity (the microscope images appear darker in the corners). In order to do this, the arrays corresponding to the images of the samples containing a hole are divided element-wise by the array corresponding to the image taken of the calibration sample at an externally applied strain of 0%. Prior to this operation, the calibration image is smoothed with a 2D Gaussian filter, in order to avoid introducing additional noise in the strain map (imgaussfilt). The local strain is calculated from the array of corrected intensity values element-wise using the following equation obtained from the exponential fit on the calibration data: $\varepsilon_{local} = \log((I_{corr}-y_{0,calib})/A_{calib})/R_{0,calib}$. Once the array of local strain values is obtained, two corrections are applied. Firstly, the value of I_{corr} in some of the array elements is less than the y_0-value obtained from the calibration. This means that the script calculates the log of a negative number, resulting in a strain value that is a complex number. An operation is performed to set these elements to "NaN", which allows them to be disregarded in the analysis routine, while maintaining the dimensions of the array. Secondly, a local strain value of less than 0 is sometimes obtained. These elements are also disregarded in the analysis, as the calibration was performed in tension only, and it cannot be assumed that a similar log relationship between intensity and applied strain exists in compression. From this point on in the script, the calculation of the local strain profile is the same as for the **tOPV** and **Loop** samples.

The micrographs obtained from the confocal microscope are grayscale 16-bit 1024 × 1024 two-page tiff stacks. The first page in the tiff stack corresponds to the micrograph taken with the green filter, and the second to the micrograph taken with the red filter. These are first read into MATLAB (imread), and converted to a numerical array with double precision which is necessary for the following numerical manipulation (im2double). As for the **Loop** data obtained with the widefield fluorescence microscope, the array corresponding to the green fluorescence micrograph is divided element-wise by the array corresponding to red fluorescence micrograph (bsxfun). The I_M/I_E ratios obtained in each pixel of this array are then used to calculate local strain values, using the parameters obtained from the calibration. These parameters are the slope and intercept of a straight line fit to $\Delta(I_M/I_E)$ vs. externally applied strain.

For the calibration sample, $\Delta(I_M/I_E)$ is calculated by subtracting the average value of I_M/I_E at an applied strain of 0% (i.e. $I_{M,0}/I_{E,0}$) from I_M/I_E for all the strain-points. For the sample containing the glass beads, a similar approach was employed, but $I_{M,0}/I_{E,0}$ was calculated by taking the average I_M/I_E on a part of the sample at 0% applied strain away from the beads in the top left-hand corner of the image. A first local strain map is then visualised for inspection (imagesc). Thresholding is used to identify the locations of the glass beads and the cavities around them. The green image is segmented into four levels of intensity (imquantize), and those parts of the image belonging to lowest level of intensity are defined as the defects (beads and cavities). A mask array is generated where the defects are defined as '0' and the surrounding matrix as '1'. White spaces within the black holes are filled in (imfill). The script removes holes with an area of less than 1500 pixels (bwareaopen), corresponding to approximately to a glass bead with a diameter of 6 μm (given a reported diameter of 9-13 μm), from the list of identified defects. The number of identified defects is calculated (regionprops, numel) and the (x,y)-coordinates of the pixels in the boundaries of the areas corresponding to the defects are extracted (bwboundaries).

The local strain profile around each defect is then calculated and plotted on the local strain map in a for loop which runs over the set of identified defects. In this loop, duplicate points are first removed from the boundary. The centroid of the hole is calculated from the averages of the x- and y-coordinates of the points on the edge of the hole area. The path along which the strain profile is extracted is defined at a constant distance of 20 pixels away, corresponding to a distance of 2.6 μm, from the edge of this area (polybuffer), and plotted on the local strain map. The points from the profiles which are located beyond the edges of the micrograph are removed, and the local strain values are retrieved from the local strain array for the selected pixels in this path (also within a for loop). The (x,y)-coordinates are converted to polar coordinates in the manner described for the analysis of the widefield fluorescence micrographs. The MATLAB scripts are included in the online repository of the source data.

Synthetic procedures

Synthesis of Loop-PU. Loop-PU was synthesised as previously reported.[1] **Loop-PU** was obtained as a pink rubbery solid (4.0304 g, 96.5%) and characterised by ^{1}H NMR.

^{1}H NMR (400 MHz, $CDCl_3$) δ 6.34 (s, 1H), 4.14 (s, 4H), 3.64 – 3.41 (m, 30H), 3.16 (d, *J* = 7.0 Hz, 4H), 1.90 – 1.42 (m, 38H), 1.39 – 1.20 (m, 5H).

NMR Spectra

^{1}H NMR spectrum ($CDCl_3$, 400 MHz) of **Loop-PU**.

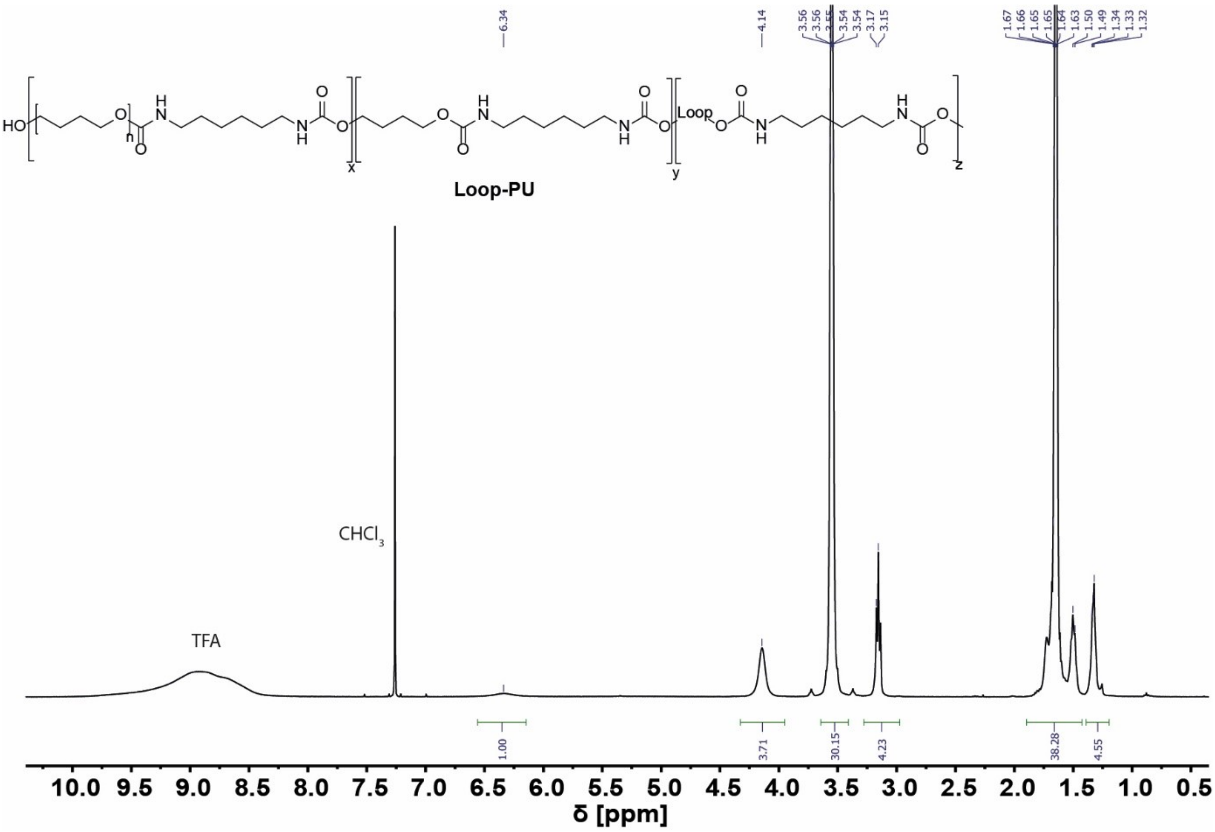

Literature

1 H. Traeger, Y. Sagara, J. A. Berrocal, S. Schrettl and C. Weder, *Polym. Chem.*, 2022, **13**, 2860–2869.

2 C. Calvino, Y. Sagara, V. Buclin, A. P. Haehnel, A. del Prado, C. Aeby, Y. C. Simon, S. Schrettl and C. Weder, *Macromol. Rapid Commun.*, 2019, **40**, 1–8.

3 D. J. Kiebala, Z. Fan, C. Calvino, L. Fehlmann, S. Schrettl and C. Weder, *Org. Mater.*, 2020, **02**, 313–322.

4 Y. Sagara, M. Karman, A. Seki, M. Pannipara, N. Tamaoki and C. Weder, *ACS Cent. Sci.*, 2019, **5**, 874–881.
